# NanoBRET in *C. elegans* illuminates functional receptor interactions in real time

**DOI:** 10.1186/s12860-022-00405-w

**Published:** 2022-01-31

**Authors:** Victoria Elisabeth Groß, Miron Mikhailowitsch Gershkovich, Torsten Schöneberg, Anette Kaiser, Simone Prömel

**Affiliations:** 1 Rudolf Schönheimer Institute of Biochemistry, Medical Faculty, Leipzig University, 04103 Leipzig, Germany; 2 Institute of Cell Biology, Department of Biology, Heinrich Heine University Düsseldorf, 40225 Düsseldorf, Germany; 3 Institute of Biochemistry, Faculty of Life Sciences, Leipzig University, 04103 Leipzig, Germany

**Keywords:** NanoBRET, *C. elegans*, Receptor-ligand interaction, Enhanced bystander BRET

## Abstract

**Background:**

Protein-protein interactions form the basis of every organism and thus, investigating their dynamics, intracellular protein localization, trafficking and interactions of distinct proteins such as receptors and their ligand-binding are of general interest. Bioluminescence resonance energy transfer (BRET) is a powerful tool to investigate these aspects in vitro. Since in vitro approaches mostly neglect the more complex in vivo situation, we established BRET as an in vivo tool for studying protein interactions in the nematode *C. elegans.*

**Results:**

We generated worms expressing NanoBRET sensors and elucidated the interaction of two ligand-G protein-coupled receptor (GPCR) pairs, the neuropeptide receptor NPR-11 and the Adhesion GPCR LAT-1. Furthermore, we adapted the enhanced bystander BRET technology to measure subcellular protein localization. Using this approach, we traced ligand-induced internalization of NPR-11 in vivo.

**Conclusions:**

Our results indicate that in vivo NanoBRET is a tool to investigate specific protein interactions and localization in a physiological setting in real time in the living organism *C. elegans*.

**Supplementary Information:**

The online version contains supplementary material available at 10.1186/s12860-022-00405-w.

## Background

The investigation of protein-protein interactions is of great interest to scientists of many fields since they are crucial for most physiological processes and thus, also represent a focus in drug discovery. Monitoring interactions, protein localization and intracellular trafficking has become increasingly feasible in the past years with the development of several tools, among others bioluminescence resonance energy transfer (BRET) (summarized in [1, 2]). BRET relies on a donor emitting bioluminescence and a fluorescent acceptor. The donor is usually an enzyme catalyzing the reaction of a luminogenic substrate. In this reaction, light is emitted and the energy is transferred to a suitable acceptor in close proximity (< 10 nm), which emits fluorescence upon excitation. These “tags” are used to study the interaction of proteins when they are fused to them. Common BRET pairs are luciferases and fluorescent proteins such as the green fluorescent protein (GFP) [3, 4]. The extensive use of BRET led to a continuous optimization of the system. The newest achievement is the small engineered Nanoluciferase (Nluc), which is much smaller (19 kD) than the widely used Rluc from *Renilla reniformis* (36 kD) or the firefly Fluc (61 kD), making it ideal for fusions with other proteins. Further, it provides a 150-fold higher/more intense luminescence than other luciferases [5]. The resulting BRET is referred to as NanoBRET [6] and is more sensitive and versatile than BRET approaches using Rluc or Fluc.

BRET has been very successfully employed to investigate general protein-protein interactions in living cells (reviewed in [6]), intracellular trafficking [7, 8], conformational changes [9] and dimerization [10, 11] of proteins. One main area of BRET applications is the investigation of ligand-receptor binding (reviewed in [2, 12]). To date, BRET studies are mainly restricted to cell culture. The use of this method in animal models is rare for several reasons such as poor tissue accessibility or signal intensity and duration and more laborious manageability (summarized in [13]). However, application of the BRET technology in vivo offers the opportunity to study protein interactions or ligand-receptor binding in a more complex cellular environment. This is especially of advantage when co-factors or other, unidentified molecules are required to e.g. stabilize ligand-binding. These might not be present in a cell-based environment, but are naturally present in the endogenous setting of the proteins to be studied. Thus, special BRET approaches closer to in vivo settings have been used to monitor for instance tumor growth in mice, where luciferase-expressing cells were injected into the animals [14–17]. Furthermore, BRET is traceable also in vivo by co-injecting fluorescent ligands or fusion proteins [18, 19]. Nevertheless, in vivo BRET studies in animals directly expressing a luciferase as a BRET donor do not exist to our knowledge, including common model organisms such as fish (*D. rerio*), fly (*D. melanogaster*) or worm (*C. elegans*). However, these animals are not only suitable models for many physiological processes, but also bear many advantages such as being small and easily cultivated. Thus, BRET studies would be highly beneficial to address questions on protein-protein interactions, protein trafficking, subcellular localization, and ligand-binding of receptors in a broader cellular and organismal context.

Among these model organisms, the nematode *C. elegans* stands out as most suitable to implement the BRET method in vivo since it is completely translucent and easy to genetically manipulate. Measuring bioluminescence as a marker, for example for ATP concentrations [20] or gene expression [21], is already a common tool and the use of fluorescent markers is also well established in *C. elegans* [22].

Therefore, our aim was to establish specific NanoBRET sensors in *C. elegans* for the investigation of protein-protein interactions as well as protein localization in vivo. As receptor-ligand binding studies are to date the most common fields of application for BRET, we chose two very different G protein-coupled receptors (GPCR) and their ligands: the neuropeptide receptor NPR-11 with its ligand FLP-34-1 and the Adhesion GPCR LAT-1 with a synthetic peptide derived from a tethered agonistic sequence buried within the receptor. NPR-11 possesses the closest sequence and pharmacological similarities to the well-characterized human neuropeptide Y (NPY)/neuropeptide F (NPF) system [23], which is conserved in almost all bilaterians and has essential physiological functions in regulating food uptake and learning (reviewed in [24–27]). NPR-11 is activated by several neuropeptides, among others by FLP-34-1, which has conserved structural motifs similar to NPY [23, 28].

The second GPCR, the Adhesion GPCR LAT-1 is one worm homolog of Latrophilins and has neuronal (summarized in [29]) and developmental [30, 31] functions. The receptor is activated by a tethered agonist sequence (termed *Stachel*), which is located within the N terminus of the receptor [32]. Peptides derived from this sequence are also able to activate LAT-1 [31].

To enable BRET-based ligand binding assays, both receptors were fused to a Nanoluciferase (Nluc) [5] at their N terminus serving as energy donor. Their corresponding ligands were tagged with the fluorescent 5(6)-carboxytetramethylrhodamine (TAM) as energy acceptor. Using this assay, we show interaction for both ligand-receptor pairs, but highlight that low-affinity binding is difficult to determine in this in vivo system.

Additionally, we have adapted and tested an enhanced bystander NanoBRET assay [8] to elucidate subcellular localization and recycling of NPR-11, which relies on spatial proximity between a plasma membrane marker based on an mNeonGreen (mNG) fluorophore fused to the CAAX-targeting sequence (polybasic sequence containing a prenylation sequence), and NPR-11 fused C-terminally to the Nluc. This revealed a temporal reduction of NPR-11 in the plasma membrane after stimulation with its ligand FLP-34-1, suggesting receptor internalization.

## Results

### Luminescence of a BRET donor-fusion protein depends on the protein of interest and nematode condition

**Fig. 1:**
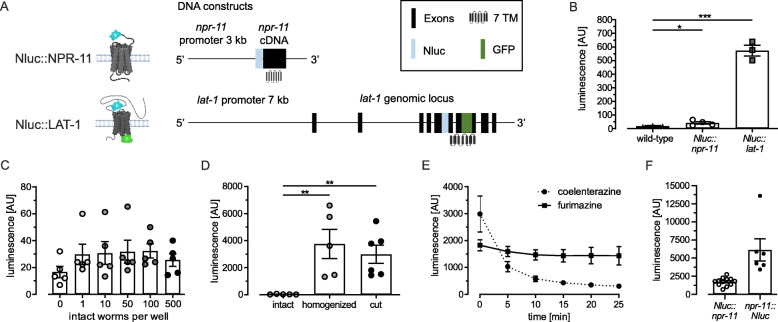
Different treatments enhance substrate availability and luminescence detection in Nluc-expressing nematodes. **A** Schematic representation of constructs used in this study. For luminescence detection and subsequent BRET analyses, a Nanoluciferase (Nluc) fused to either NPR-11 or LAT-1, respectively, was expressed in the respective knockout nematodes. A GFP was fused to the second intracellular loop of Nluc::LAT-1 for monitoring correct localization of the protein at the cell membrane. Synchronized young adult nematodes (L4 + 1 day) were left intact or cut, and luminescence was directly measured after coelenterazine H addition. **B** In intact wild-type nematodes, basal luminescence is 19.5 ± 2.2 AU, while it is 2-fold higher in *Nluc::npr-11* worms. Luminescence of *Nluc::lat-1* is 14-fold higher than in *Nluc::npr-11* worms. **C** Luminescence of Nluc::NPR-11 in intact worms is not increasing when raising the animal counts per well. **D** *Nluc::Npr-11* worms homogenized with beads and harsh shaking provide a strong luminescence signal compared to intact animals. Luminescence of nematodes with an incision is comparable to homogenized samples. **E** Luminescence in cut worms incubated with coelenterazine decreases over time (− 90% from 0 min to 25 min) while furimazine provides stable values over the entire time period. F Luminescence in cut worms either expressing *Nluc::npr-11* or *npr-11::Nluc* incubated with furimazine. Both strains show luminescence. Data in **B**-**F** are shown as mean ± SEM; *n* ≥ 3, *N* = 50 with 3 technical replicates; *p ≤ 0.05; **p ≤ 0.01; ***p ≤ 0.001; two-sided t-test; AU = arbitrary units. Schematic pictures were created with BioRender.com

One essential pre-requisite for a stable BRET signal is a bright and constant luminescence signal of the donor. In order to establish BRET analyses in *C. elegans*, we first sought to optimize the luminescence signal intensity emitted from the donor, which is highly dependent on the accessibility of the donor itself and the availability of the substrate converted by the donor. To ensure these aspects, we generated nematodes stably expressing a Nanoluciferase (Nluc) [5] as donor fused to the extracellular N terminus of NPR-11 (Nluc::NPR-11, the strain is referred to as *Nluc::npr-11* as well as the transcript) and LAT-1 (Nluc::LAT-1, the strain is termed *Nluc::lat-1* as well as the transcript), respectively (Fig. 1A). A SGGGGS linker provided some conformational flexibility of the Nluc. Expression of these constructs was confirmed by fluorescence confocal microscopy. It has to be noted that expression pattern of *npr-11* is not as complete as described by others [28]. This is most likely because the construct used in our study contains a 3 kb promoter. A longer sequence could yield a broader expression pattern.

While the fluorescence of Nluc::NPR-11::GFP was limited to two cells (presumably neurons) within the nematode tail (Fig. S1A), *Nluc::lat-1::GFP* expression was detected around the membrane of pharyngeal muscles (Fig. S1B).

Different set-ups were assessed to determine the one providing the most intensive and stable luminescence signal while at the same time keeping the nematodes as intact as possible and the number of animals to a minimum. To enable a reliable quantification, these analyses were conducted using a plate reader. First, we studied the luminescence intensity of 50 synchronized intact, viable animals (L4 + 1 day). A signal was detected in both strains, *Nluc::npr-11* and *Nluc::lat-1*, which was significantly increased compared to the one of the wild-type after incubation with the substrate coelenterazine H (Fig. 1B). The luminescence of Nluc::LAT-1 was 14-fold higher than the signal of Nluc::NPR-11, probably due to the difference in expression levels and patterns of both receptors. The signal of the *Nluc::npr-11*-expressing nematodes was only slightly above background (Fig. 1B), yielding suboptimal conditions for further analyses. Thus, we sought to optimize the luminescence signal of this strain to make the technique amenable also for lowly expressed genes. Firstly, we altered the number of animals per well to check whether this correlates with luminescence intensity (Fig. 1C). However, increasing amounts of worms did not render a higher luminescence signal. The reason for this might be that blue-shifted light is scattered by tissue [33], thus perturbing any increase in signal, or limited diffusion of the substrate across the cuticle.

Secondly, we aimed at elevating substrate availability within a sample. Sfarcic et al. have previously reported that detection of luminescence is greatly increased in lysed worms [21]. Thus, we ruptured the nematodes to facilitate substrate distribution. As we aimed at keeping the cells as intact and viable as possible, we compared two different approaches: homogenization, and making an incision with a scalpel (Fig. 1D). Both techniques significantly increased the luminescence up to 3000–4000 AU (Fig. 1D). As cutting an incision kept the worms mostly intact and proved to provide sufficient luminescence signal, all following analyses in this study were conducted using the incision method to prepare worms.

Finally, we evaluated whether the luminescence signal can be improved by using a different substrate. Coelenterazine H lost its activity over time when used on worms, reaching 10% of the initial value after 25 min (Fig. 1E). Therefore, an alternative substrate optimized for brightest luminescence with the nanoluciferase, furimazine [5], was tested, resulting in a lower but more stable luminescence signal (Fig. 1E). Thus, for subsequent studies, assays were performed with furimazine. Further, we verified that furimazine enters the cells in the worm by assaying a strain expressing *npr-11::Nluc* and found that the luminescence signal is not weaker than in the strain *Nluc::npr-11* carrying the Nluc at the extracellular N terminus of NPR-11 (Fig. 1F).

Taken together, our analyses showed that bioluminescence can be reliably measured in small quantities of nematodes expressing Nluc fused to proteins. Signals from proteins with lower expression levels can be analyzed when using the incision method to prepare the worms and furimazine as substrate. These data formed to basis for subsequent BRET analyses.

### Tetramethylrhodamine-labeled peptides as BRET acceptors

Besides the energy donor, an acceptor is required in order to enable BRET analyses. Peptide-activated GPCR are highly suitable for BRET studies because both receptors and ligands can be easily labeled. Furthermore, receptors are well-accessible at the cell surface and peptides can be applied in a relatively simple manner compared to other ligands.

**Fig. 2:**
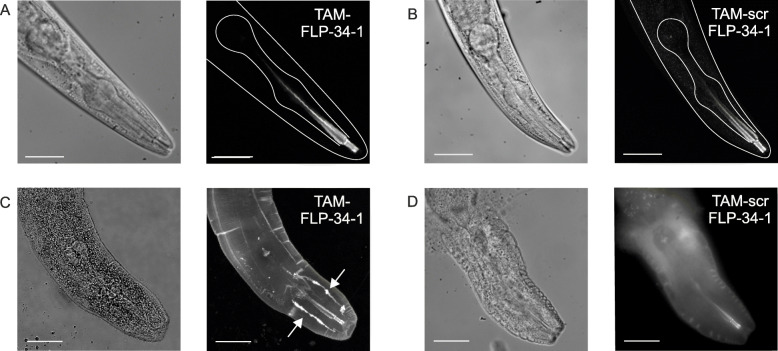
Accessibility and distribution of TAM-FLP-34-1 and TAM-scrFLP-34-1 in the heads of wild-type nematodes. **A, B** Representative fluorescence microscopy images of intact wild-type nematodes (L4 + 1 day) incubated for 10 min with a tetramethylrhodamine (TAM)-labeled version of the natural NPR-11 ligand, FLP-34-1 (5 μM) (**A**), and its corresponding scrambled (scr) version, TAM-scrFLP-34-1 (5 μM) (**B**). Fluorescence of both peptides in intact worms is only visible within the lumen of the pharynx (left: brightfield, right: fluorescence channel). **C** TAM-labeled FLP-34-1 in worms with incisions shows a specific pattern of distribution, such as in the pseudocoelom (white arrows). **D** The corresponding scrambled (scr) version TAM-scrFLP-34-1 spreads evenly, but does not accumulate at certain cells or structures. Please note that the integrity of the worms in **C** and **D** is damaged due to the procedure influencing the morphology of the structure. Scale bars = 50 μm. *n* = 4 (*N* ≥ 8) with 76% of the investigated worms showing the depicted pattern

We labeled the peptide ligands of NPR-11 and LAT-1 (FLP-34-1 for NPR-11 and pLAT-1 for LAT-1) with the fluorescent tetramethylrhodamine (TAM), which served as energy acceptor and fluorescent tracer. To check that these TAM-labeled peptides penetrate the tissue and distribute equally, the fluorescence signal in pre-treated intact worms and in nematodes with an incision was determined (Fig. 2, Fig. S2). For that purpose, the TAM label was directly excited and fluorescence detected in the heads of animals treated with either TAM-FLP-34-1 or a scrambled control version (TAM-scrFLP-34-1, random composition of the same amino acids resulting in comparable chemical properties). While in intact worms, fluorescence was only detectable within the lumen of the pharynx up to the terminal bulb for both peptides (Fig. 2A, B), it was spread in the entire pseudocoelom and even accumulated on some structures in worms with incisions incubated with TAM-FLP-34-1 (Fig. 2C, white arrows). These patterns did not appear during the incubation with TAM-scrFLP-34-1 (Fig. 2D), indicating a specific distribution of TAM-FLP-34-1. TAM-pLAT-1 showed a similar pattern (Fig. S2A, C), however, no difference to TAM-scrpLAT-1 was detected (Fig. S2B, D). These results indicate that TAM-labeled peptides do not enter the nematodes properly when simply fed, but penetrate worms with incisions.

Next, we verified that the TAM label does not disturb binding of the peptides and their capability to activate NPR-11. As the conserved bioactive part of FLP neuropeptides resides in their C terminus [23, 34], any modifications to the free N terminus of FLP-34-1 were expected to be tolerated. This proved to be the case when we elucidated the activity in cell culture using a NanoBRET assay for elucidating binding properties and a cAMP reporter gene assay for analyzing activation (Fig. S3). It was previously shown that FLP-34-1 is a potent endogenous ligand of NPR-11 [23, 28]. In our NanoBRET assay set-up, adding TAM-FLP-34-1 in increasing concentrations to Nluc::NPR-11 led to the formation of a specific BRET window of 0.29 ± 0.01(difference between donor-only and donor/acceptor values, i.e. unstimulated/stimulated samples) with a K_d_ value of 388 nM. TAM-scrFLP-34-1 yielded no signal above baseline levels up to concentrations of 3 μM (Fig. S3A). These data are consistent with previously performed competition-binding assays using TAM-FLP-34-1 as fluorescent tracer, in which unmodified FLP-34-1 displays an inhibition binding constant K_i_ of 107 nM [35]. This is comparable to the K_d_ value of the fluorescently labeled peptide, and thus, corroborates the wild type-like properties of TAM-FLP-34-1.

To evaluate the ability of the TAM-labeled peptides to activate NPR-11, a cAMP reporter gene assay was performed. Activity of GPCR is detectable through their binding to G proteins and the subsequent change in second messenger concentrations. Since NPR-11 couples to G_i_ proteins, pre-stimulation with forskolin (an activator of the cAMP-producing adenylyl cyclase) was necessary to increase intracellular cAMP levels and measure the ability of NPR-11 to inhibit this process. Changes in intracellular cAMP levels can be determined by a reporter gene assay. In this assay, TAM-FLP-34-1 showed the capability to activate NPR-11 (EC_50_ = 0.5 nM) similar to the one of the unmodified peptide (EC_50_ = 2.3 nM), while the scrambled peptide (TAM-scrFLP-34-1) did not activate NPR-11 (Fig. S3B) and served as negative control.

To activate LAT-1, we used a synthetic peptide derived from the tethered agonist sequence [31]. To generate a fluorescently-labeled version (TAM-pLAT-1), the TAM fluorophore was attached in the C-terminal part of the peptide to minimize functional interferences as it has been shown that the free N-terminal threonine is important for the agonistic properties of the peptide [36]. A cAMP accumulation assay revealed that TAM-pLAT-1 harbored the same activity as unlabeled pLAT-1 (cAMP accumulation 1.5-fold over control at a concentration of 100 μM, Fig. S3C). The respective scrambled peptide TAM-scrpLAT-1 did not lead to a cAMP accumulation at the same concentration (Fig. S3C).

These data clearly indicate that TAM labeling did not alter binding or activity of the peptides they are conjugated with and thus, they can be employed for in vivo BRET analyses.

### The interaction between TAM-labeled peptides and Nluc-fused receptors can be monitored by BRET

**Fig. 3:**
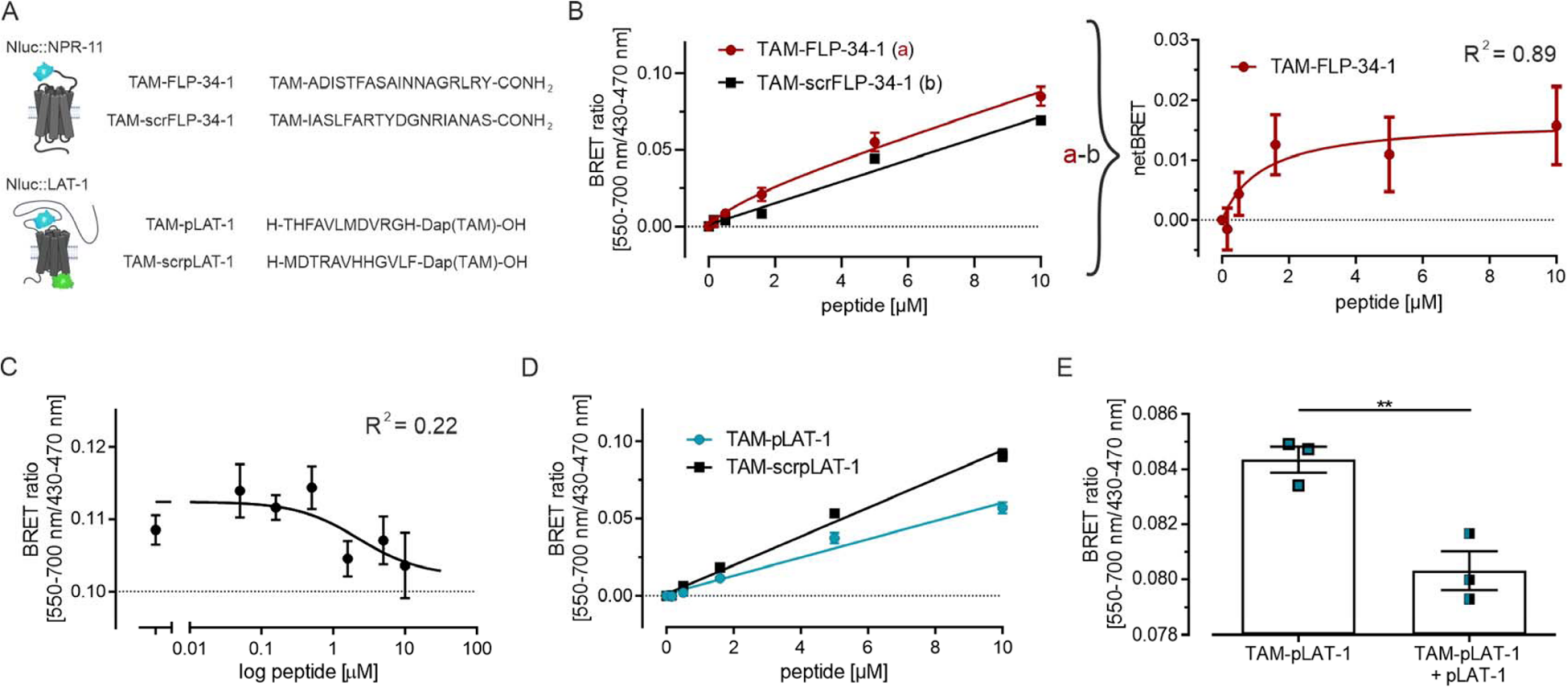
Affinity and specificity of TAM-labeled peptides at their corresponding receptors. BRET binding assays were performed to determine the binding of TAM-labeled peptides to the Nluc-fused receptor with slightly incised Nluc-expressing worms after 25 min incubation. **A** Schematic depiction of Nluc::NPR-11 (top, left) and Nluc::LAT-1 (bottom, left) with the amino acid sequence of the respective TAM-labeled FLP-34-1 and pLAT-1 and their corresponding scrambled (scr) versions (right). In TAM-pLAT-1 and TAM-scrpLAT-1, the fluorophore is attached to the side chain of the additional amino acid Dap (diaminopropionic acid). **B** TAM-FLP-34-1 binding to Nluc::NPR-11 shows a BRET window with a K_d_ value of 1.4 μM (left panel). Although there is no significant difference between the values from a) and b) (two-way ANOVA), the specific window appears clearly when calculating the netBRET between agonistic and scrambled control peptide (right panel). **C** Displacement assay based on NanoBRET. 1.6 μM TAM-FLP-34-1 were displaced with increasing concentrations of unlabeled FLP-34-1 at Nluc::NPR-11. FLP-34-1 is able to displace TAM-FLP-34-1 with a K_i_ value of 1.1 μM. The background signal from filter overlap is 0.10 and is indicated as dashed line, representing complete displacement. **D** TAM-pLAT-1 binding to Nluc::LAT-1 generates a BRET window, which is even larger with TAM-scrpLAT-1. This BRET signal was not saturable up to 10 μM, and thus, the K_d_ was estimated to be > 10 μM for both peptides. **E** NanoBRET of Nluc::LAT-1 incubated with TAM-pLAT-1 (0.5 μM) without and with 10 μM unlabeled pLAT-1 was determined after 60 min incubation showing that pLAT-1 is able to displace TAM-pLAT-1. Depicted in **B**-**E** is the mean ± SEM of n ≥ 3 assays with *N* = 30 worms in triplicates; ** p ≤ 0.01; two-sided t-test. Data in **B** and **D** are baseline-corrected to the respective 0 μM value. Luminescence and fluorescence values are given in Table S1. Note that calculation of BRET ratios are based on the raw luminescence and fluorescence values of each performed experiment. For these values, see Table S4. Schematic images were created with BioRender.com

Based on the findings, settings and approaches described above, an experimental set-up was established to measure BRET in vivo in *C. elegans*. BRET pairs were generated between the Nluc-fused receptors (Nluc::NPR-11; Nluc::LAT-1) and their ligands (FLP-34-1; pLAT-1) or scrambled versions (scrFLP-34-1; scrpLAT-1) conjugated with TAM (Fig. 3A). In these experiments, the number of animals per well was reduced to 30 as the luminescence intensity was still sufficient (> 500 AU) to achieve a measurable BRET window (Table S1).

Synchronized nematodes (L4 + 1 day) with an incision expressing *Nluc::npr-11* were treated with TAM-FLP-34-1 and a robust BRET signal was observed. We also detected a BRET signal when using the scrambled control peptide TAM-scrFLP-34-1 (Fig. 3B). However, the increase in the BRET ratio by TAM-FLP-34-1 was higher than by TAM-scrFLP-34-1 (TAM-FLP-34-1 = 0.08 ± 0.012; TAM-scrFLP-34-1 = 0.07 ± 0.003; at 10 μM) (Fig. 3B, left panel). To calculate the specific binding of TAM-FLP-34-1 to Nluc::NPR-11, we treated the BRET signals of the scrambled peptide control as nonspecific binding (cf. Fig. S3, which shows that the scrambled version of FLP-34-1 has no measurable affinity and activity to NPR-11 in vitro), and calculated the netBRET by subtracting the TAM-scrFLP-34-1 BRET ratio from the respective TAM-FLP-34-1 BRET ratio. This showed that a specific BRET window opens starting from around 1 μM peptide concentration, which was saturable with increasing concentrations (Fig. 3B, right panel). Accordingly, the K_d_ value of TAM-FLP-34-1 in vivo was calculated to be 1.4 μM, which was 4-fold higher, but still in reasonable agreement with the K_d_ value determined in vitro (in vitro: 388 nM). We assume that the BRET effect of the scrambled control peptide is a result of the more complex in vivo situation, in which this peptide variant might bind to other protein targets in the vicinity of NPR-11, thus causing BRET without directly interacting with NPR-11.

To substantiate that binding of TAM-FLP-34-1 to Nluc::NPR-11 is specific, a displacement binding study was conducted, in which the amount of unlabeled FLP-34-1 was increased while the concentration of TAM-FLP-34-1 remained constant. FLP-34-1 displaced TAM-FLP-34-1 with a K_i_ value of 1.1 μM indicating a specific binding of the peptides (Fig. 3C). Taken together, stable NanoBRET is generated while endogenously expressing the Nluc-fused *npr-11* and administering TAM peptides.

To further investigate the potential of NanoBRET in *C. elegans*, we assessed the second receptor-peptide pair, Nluc::LAT-1 and pLAT-1, which has a reportedly lower potency and typically requires approximately 1 mM peptide to activate the receptor [31]. Incubation of *Nluc::lat-1*-expressing worms with TAM-pLAT-1 generated robust BRET windows that increased with rising peptide concentrations (Fig. 3D). Surprisingly, the scrambled control TAM-scrpLAT-1, which proved inactive in in vitro signaling assays (Fig. S3C), provided an even stronger increase in BRET signal with a one-third higher BRET ratio at 10 μM than TAM-pLAT-1. In both cases, the BRET signal was not saturable up to 10 μM, and thus, the K_d_ value was estimated to be > 10 μM (Fig. 3D). Similar to the situation with the scrambled version of FLP-34-1 described above, we assumed that the BRET signal of the scrambled pLAT-1 originated from binding to proteins in the vicinity of LAT-1, or attaching to other sites in LAT-1. The presumably low binding affinity of pLAT-1 prevented the calculation of specific binding by subtracting the signal of the scrambled peptide in this case. However, the different BRET windows for TAM-pLAT-1 versus TAM-scrpLAT-1 suggest that the peptides occupy different sites, and TAM-pLAT-1 might be enriched at a site with less efficient BRET compared to random orientations in unspecific binding of TAM-scrpLAT-1.

To gain further information on the specificity of TAM-pLAT-1, the ability of unlabeled pLAT-1 to displace TAM-pLAT-1 was monitored (Fig. 3E). After 60 min of incubation, we found a partial reduction of the BRET signal in the presence of excess unlabeled pLAT-1. These data suggest that partial displacement is occurring.

To conclude, these data show that NanoBRET measurements are feasible to estimate binding affinities in vivo within the nematode, and work particularly well for high-affinity interactions with K_d_ values up to the low micromolar range.

### Enhanced bystander BRET in vivo reveals potential internalization of NPR-11

**Fig. 4:**
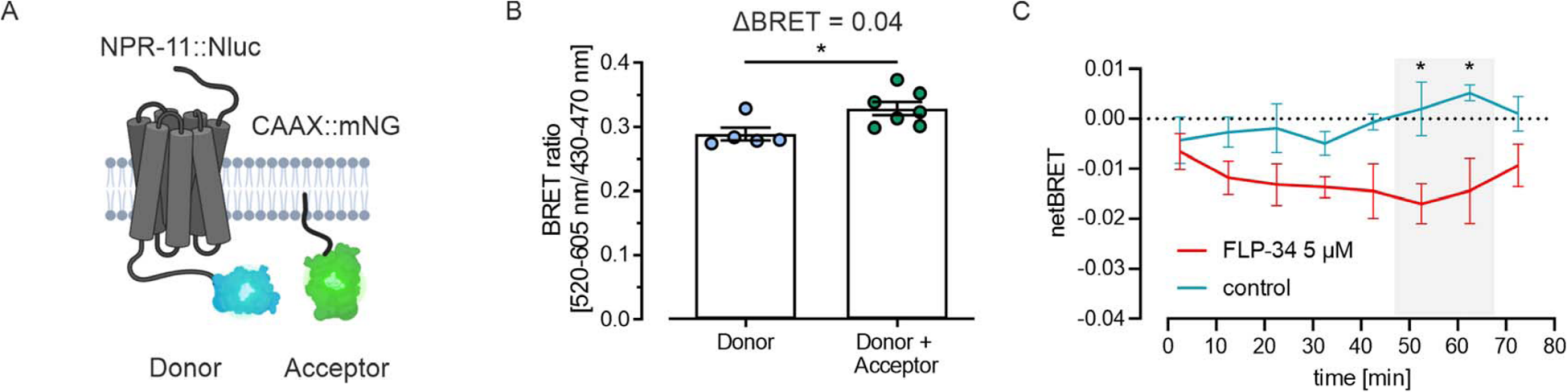
Enhanced bystander BRET reveals information on receptor trafficking and internalization of NPR-11 in vivo. **A** Bystander BRET was determined between Nluc fused to the C terminus of NPR-11 (NPR-11::Nluc, donor) and an mNeonGreen fused to the C-terminal part of let-60 (CAAX::mNG, acceptor) expressed in the same individual. **B** Donor and acceptor are generating a bystander BRET signal that is significantly higher than the donor-only (baseline) BRET ratio. Experiments were set-up as in Fig. 3. Luminescence and fluorescence values are given in Table S1. Shown is the mean ± SEM in *n* ≥ 5 assays with 30 worms per well as triplicates; * *p* ≤ 0.05 two-sided t-test. **C** Enhanced bystander BRET (measured over time) of worms treated either with BSA or 5 μM FLP-34-1, baseline-corrected (value - baseline) for every single time point and pruned rows (mean of every 10 min value with the following value starting with t = 2.5 min). While the control stays stable around the baseline, the netBRET of FLP-34-1 drops after 42.5 min, opening a significant window till 62.5 min (grey area), suggesting a separation of donor and acceptor. Shown is the mean ± SEM in *n* = 3 assays with 30 worms per well as triplicates; * p ≤ 0.05 two-way ANOVA, Bonferroni‘s posthoc test. Schematic images were created with BioRender.com

Besides direct interactions, the subcellular localization of proteins as well as the proximity to other proteins are of great interest. In vitro studies do not completely reflect the situation in vivo, since the proteins are not in their natural environment. Therefore, we adapted the enhanced bystander BRET methodology in vivo to examine subcellular localization of proteins. Here, NPR-11 C-terminally fused to the Nluc was chosen as energy donor while the green fluorescent protein mNeonGreen (mNG) fused to the CAAX motif from *let-60* served as energy acceptor (Fig. 4A). *let-60* encodes for a human K-Ras homolog (GTPase) present at the plasma membrane. The CAAX motif serves as a plasma targeting sequence and prenylation signal, ensuring correct localization of a protein at the plasma membrane (summarized in [37]). We generated a worm strain stably expressing mNeonGreen at the plasma membrane of all cells (expression driven by the strong ribosomal promoter *rpl-28p*) (schematic representation in Fig. 4A, expression in Fig. S4). Enhanced bystander BRET was detected by comparing the donor-only-expressing strain with worms harboring both, donor and acceptor. The donor-only strain provided a stable ‘BRET’ baseline around 0.28 ± 0.01, which was the experimental background due to filter overlap and potential induction of background fluorescence in the cellular background. In the presence of donor and acceptor, the BRET signal was elevated to 0.32 ± 0.01 (Fig. 4B), demonstrating that a productive BRET is occurring due to the proximity of the receptor and plasma membrane marker, yielding a net measurement window of approximately 0.04 BRET units.

**Fig. 5:**
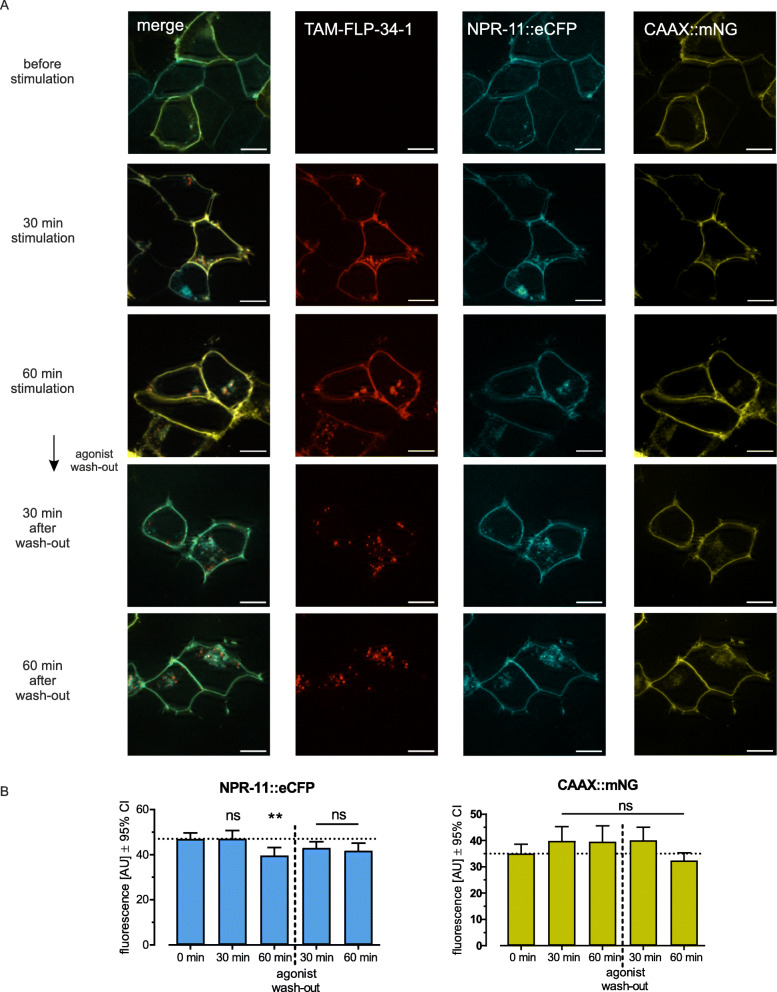
Enhanced bystander BRET in vitro shows potential NPR-11 internalization. **A** NPR-11 might undergo agonist-promoted endocytosis in vitro while the membrane marker remains at the plasma mebrane. A genetic fusion of NPR-11 with a cyan fluorescent protein (eCFP) and a mNeonGreen anchored to the plasma membrane via the CAAX motif was expressed heterologously in HEK293 cells. Cells were stimulated with 100 nM TAM-FLP-34-1, subcellular localization was monitored in live cells after 30 min, 60 min, and additionally 30 min and 60 min after agonist wash-out. Cell nuclei were stained with Hoechst33342. **B** Membrane fluorescence measurement (x-fold of before stimulation) of NPR-11::eCFP (left panel) and CAAX::mNG (right panel) before stimulation, 30 min and 60 min before and after agonist wash-out with cells treated as described in A. While the fluorescence of CAAX::mNG remains stable over time, the fluorescence of NPR-11::eCFP drops significantly after 60 min of stimulation suggesting peptide-mediated internalization. Fluorescence levels recover after agonist wash-out. Shown is the mean ± SEM in *n* = 3 assays with *N* ≥ 10 cells per condition. * *p* ≤ 0.05, one-way ANOVA, Dunnett‘s post test against 0 min. Scale bars = 10 μm

**Fig. 6:**
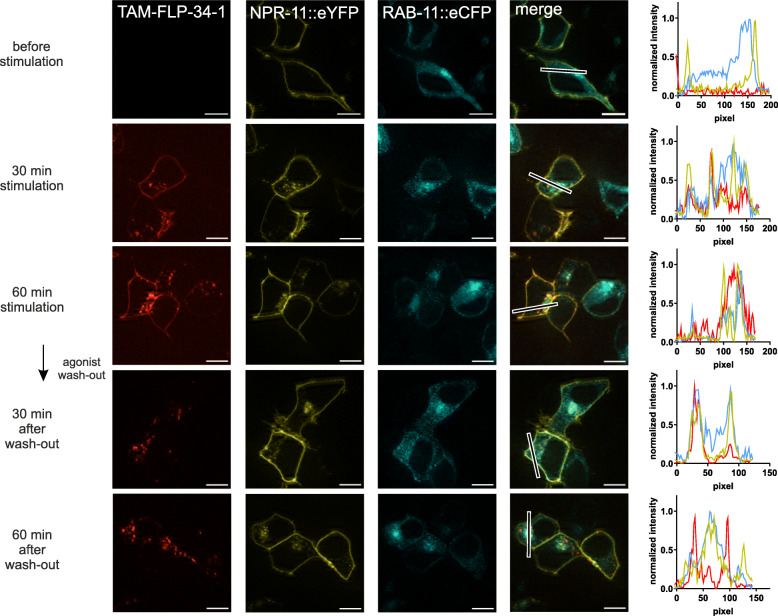
NPR-11 co-localizes with RAB11 after peptide stimulation. Potential co-localization of NPR-11 fused to an enhanced yellow fluorescent protein (eYFP) with recycling endosomes was assessed using co-expression with *rab11::eCFP* in HEK293 cells. Cells were treated as described in 5A. The panels on the right represent a quantification of the fluorescence intensities across a representative section of the merged images (black line). Scale bars = 10 μm

**Fig. 7:**
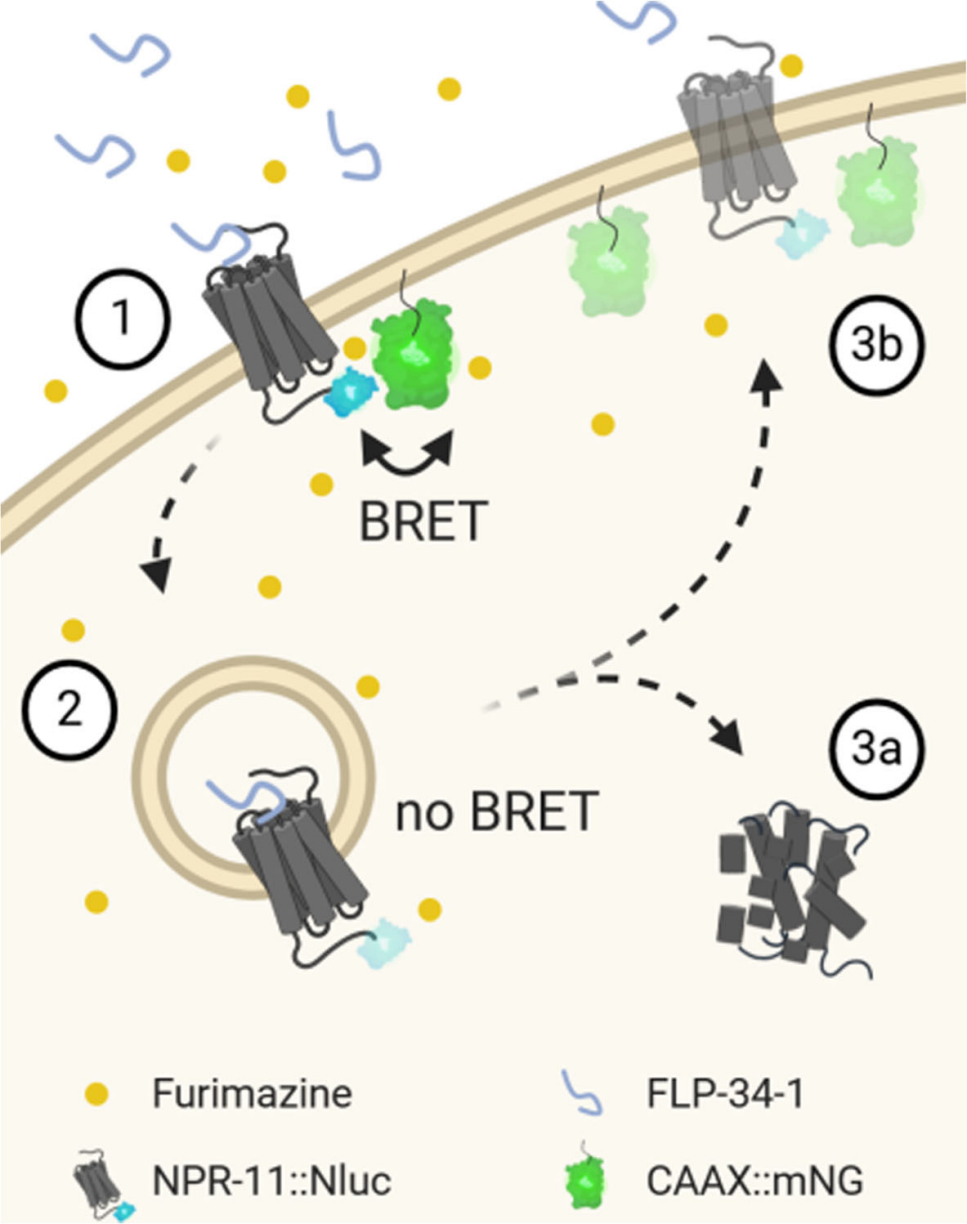
Scheme of potential receptor internalisation tracked with enhanced bystander BRET. 1 The energy donor (Nluc C-terminally fused to NPR-11) generates a bystander BRET with an energy acceptor (mNeonGreen fused to the CAAX box of *let-60*) located at the inner membrane upon furimazine application. In the presence of the agonist (FLP-34-1), the receptor undergoes endocytosis (2), lowering the number of available donor molecules at the membrane that are able to transfer energy to the acceptor. This effect shifts the ratio of luminescence and fluorescence and subsequently, yields a lower BRET window. The receptor is then either degraded in lysosomes (3a) or recycled in recycling-endosomes and transported back to the membrane. The latter one increases the bystander BRET again due to the close proximity of donor and acceptor (3b). Image created with BioRender.com

Interestingly, when treated with (unlabeled) FLP-34-1, worms carrying donor and acceptor showed a decrease of BRET signal compared to untreated controls over time (Fig. 4C). Luminescence and fluorescence were monitored over 80 min and a significant BRET window between FLP-34-1-treated and -untreated worms appeared between 50 and 70 min. This lower BRET signal in animals incubated with FLP-34-1 suggests a distancing from donor and acceptor. Since CAAX::mNG is permanently localized to the plasma membrane (Fig. S4) [8], the reduced BRET signal is likely caused by the dynamic removal of NPR-11::Nluc from the membrane upon activation. This effect reflected ligand-induced internalization followed by either receptor recycling or de novo synthesis of receptors to re-establish NPR-11 pools in the plasma membrane and was consistent with the subcellular trafficking of NPR-11 observed in vitro. To verify this observation, NPR-11 fused to an enhanced cyan fluorescent protein (eCFP) and the CAAX::mNG membrane marker were co-expressed in HEK293 cells, stimulated with its peptide ligand and receptor and marker localization were monitored by fluorescence microscopy (Fig. 5A). Before stimulation, NPR-11::CFP was present almost exclusively at the plasma membrane. 30 min after TAM-FLP-34-1 application, receptor clusters appeared in vesicular structures such as endosomes and co-localized with TAM fluorescence. Those clusters became even more pronounced after 60 min (Fig. 5A), suggesting ligand-induced internalization of NPR-11. Consistent with this observation, quantification of membrane fluorescence of NPR-11::eCFP and CAAX::mNG in the same cells confirmed receptor internalization (Fig. 5B), while CAAX::mNG remained at the plasma membrane as expected. After agonist wash-out, the receptor fluorescence slightly increased again, which might be due to receptor recycling or biosynthesis. To further investigate this effect, co-localization of NPR-11::eYFP with different cell compartments was determined. Co-localization with lysosomes was partially visible for the receptor (Fig. S5). Meanwhile, co-expression of NRP-11::eYFP with *rab11*::eCFP, a recycling endosomes marker protein [38], showed a strong overlap, in particular after agonist wash-out (Fig. 6). This led us to the conclusion that NPR-11 indeed internalized and might undergo a recycling process rather than a degradation in lysosomes (Fig. 6, Fig. S5). To conclude, we generated an enhanced bystander BRET between a cell membrane marker and the Nluc-fused receptor in *C. elegans*, forming the basis for studying subcellular protein localization and protein-protein proximity in vivo*.* Combining in vitro and in vivo results, we suggest that NPR-11 is internalized after activation, supported by the lowering BRET and fluorescent microscopy while it is rather recycled than degraded (co-localization with recycling endosomes more clearly than with lysosomes) and transported back to the membrane (increasing BRET after 70 min, schematic in Fig. 7).

## Discussion and conclusions

NanoBRET has become an indispensable tool for studying protein-protein interactions, ligand-receptor binding properties, protein trafficking, and protein localization in vitro. It yields reliable data and due to its successful application in various scientific areas, variations of BRET adapted for ex vivo studies are constantly emerging (summarized in [13]). Compared to single cells or complex model organisms such as mice, *C. elegans’* major advantage is that molecular processes can be investigated in the natural environment of proteins at a decent spatial and temporal resolution. Here, we generated specific NanoBRET sensors and established NanoBRET as a proof-of-concept for the use in the nematode *C. elegans*. We applied the method as a tool for investigating ligand-receptor interactions and highlighted that it can be adapted to different applications such as an enhanced bystander version for the study of protein trafficking in real time within a cell in the context of an entire organism. We also showed that the technique does have limitations and that careful considerations regarding the investigated interactions need to be taken.

To date, the two most considerable limitations hampering the use of NanoBRET for applications in vivo are the bioavailability of the substrate [21, 39] and the low penetrance of luminescence through tissue [33]. The small and translucent nature of *C. elegans* makes the nematode an ideal organism compared to other models to overcome these obstacles and promising studies have already reported the detection of luminescence signals in living nematodes when using the firefly luciferase/luciferin system [40–42]. In line with these studies, our data identified three parameters essential for reliably measuring luminescence in nematodes: *i)* careful selection of the substrate and the corresponding luciferase, *ii)* preparation of the animals, and *iii)* expression level of the protein fused to the luciferase. As the firefly luciferase/luciferin system is dependent on ATP and less bright than the Nluc/coelenterazine H or furimazine system, we chose the latter one for BRET analyses and furimazine as substrate due to its stability. With the set-up devised in this study, even luminescence of Nluc-fusion proteins that are present at low levels, such as NPR-11, can be detected. This was facilitated by making a small incision in the worms, which strongly increased luminescence, probably because it improves the limited bioavailability of furimazine [21, 39]. It has to be noted that due to the necessary incision of the worm, long-term studies are not possible as this incision might change the physiology and leads to premature death of the worms.

While NPR-11 is present only in a few neurons [28], the other receptor used in our study, *lat-1*, is expressed in several tissues (neurons, intestine, muscles, reproductive system [30, 43–45]), rendering much stronger luminescence signals in the respective *Nluc::lat-1* worm strain. Thus, it can be speculated that Nluc-fusion proteins that express at higher levels or abundantly, might even generate sufficient luminescence to create NanoBRET in intact worms of fewer number. It has to be noted that to increase the amount of Nluc-fused protein, we chose to use extrachromosomal expression of constructs rather than single copy integration. However, it is conceivable that single copy integrated constructs can also be detected as long as expression is sufficient.

Despite the promising feasibility of measuring luminescence and NanoBRET in *C. elegans*, the experimental set-up can be still improved. For instance, analyses of single worms are hardly possible as well as long-term studies since nematodes harmed by incisions die after a short time as mentioned above.

For assaying peptide-binding of cell surface receptors in *C. elegans* in real time, NanoBRET is a highly suitable tool. The comparison of in vivo NanoBRET with the in vitro method showed similar characteristics of the ligand-receptor binding (Fig. 3, Fig. S3). Especially for high affinity peptide-receptor interactions (K_d_ < 1 μM) as it is the case for FLP-34-1 binding to NPR-11, this proved to be true. The affinity of TAM-FLP-34-1 to the receptor is similar in both settings. The slightly higher K_d_ value in vivo (in vivo: 1.4 μM; in vitro: 388 nM) is presumably caused by differences in the effective peptide concentration directly at the receptor compared to the concentration initially applied to the medium. While in vitro*,* the peptide concentration in the medium roughly equals the concentration at the receptor, in vivo the amount of peptide reaching the receptors in the worm is most likely much lower than the initial concentration due to penetration issues. Further, the peptide amount present at NPR-11 in the nematode can be reduced as other neuropeptide receptors might be capable of sequestering the ligand (specifically as well as non-specifically) [23]. Thus, NanoBRET in vivo offers the possibility to specifically evaluate ligand-binding selectivity in a distinct tissue in the context of surrounding tissues taking aspects such as unspecific binding into consideration. Due to the clustering of different proteins in one cell, it is likely that even the scrambled peptide binds structures that are in close proximity to the Nluc, generating a certain unspecific BRET. Interestingly, the BRET windows of the in vivo measurements appear to be generally smaller that the in vitro ones (Fig. 3, Fig. S3) despite the use of the same BRET sensors and analysis filters. This effect is probably caused by the different light absorption and scattering conditions in the worm compared to single cells.

In contrast to FLP-34-1/NPR-11, the pair tethered agonist peptide/LAT-1 represents low-affinity binding. The scrambled control peptide TAM-scrpLAT-1, which does not activate the receptor (Fig. S3), still yields a BRET signal (Fig. 3). We hypothesize that the reason for this is the same as for the scrambled peptide of NPR-11, but to a stronger extent: In the environment of the cellular context, in which numerous different proteins are present, transient interactions of a peptide are likely to occur in the proximity of the receptor and may be unspecific. Thus, in this case, it cannot be determined beyond doubt that the BRET signal generated by TAM-pLAT-1 is specific and just not saturated at 10 μM, highlighting that in cases of low-affinity interactions, BRET might be not a technique of choice. However, functional analyses show that this peptide reliably activates LAT-1 (Fig. S3) [31]. Since the tethered agonist is normally directly integrated within LAT-1, low-affinity binding is sufficient for activation. This has also been shown for other Adhesion GPCR [32]. Our data show that ligand-receptor NanoBRET in vivo is a valuable tool but results need careful evaluation when studying low-affinity interactions.

It is conceivable that this in vivo NanoBRET proof-of-concept set-up can be adapted for numerous applications besides ligand-receptor interactions. Of great interest are also questions related to general protein-protein interactions and protein dimerization. The existing techniques to address these questions include analytical ultracentrifugation (reviewed in [46]), light scattering (summarized in [47]), and NanoBit (summarized in [48]). Some methods are already established in *C. elegans* such as fluorescence resonance energy transfer (FRET) [49, 50] and bimolecular fluorescence complementation (BiFC) [51, 52]. In case of the latter two, which both have been proven to be valuable and widely used techniques, protein(s) of interest are fused to fluorescent proteins or parts of them and in case of an interaction, a specific fluorescence is detected. One advantage of NanoBRET over BiFC and FRET is that no excitation with laser light is necessary and, therefore, the fluorescent background is minimized. Further, BRET offers the general possibility to measure many samples in ‘medium-throughput’ in 384-well plates in plate readers, which significantly increases throughput and facilitates handling compared to FRET experiments, which typically use microscopy.

Besides the NanoBRET technique for ligand-receptor interactions, an approach for in vivo enhanced bystander NanoBRET was established in this study yielding promising insights into protein trafficking. The cellular localization of proteins, in particular cell surface receptors, is of high interest for pharmacological interventions. For instance, in the case of GPCRs, receptor signaling from the cell membrane often is the desired pharmacologically active pathway and receptor internalization goes along with receptor desensitization. In other cases such as drug shuttling into a target cell, receptor internalization is desired, or specific signaling responses are triggered from intracellular compartments (e.g. [53, 54]).

With the CAAX::mNG we devised and employed a NanoBRET sensor for labelling the cell membrane, which can be used as a universal tool for NanoBRET assays in combination with different proteins. Our data based on this sensor showed that NPR-11 is present at the cell membrane, but the NanoBRET signal decreased after receptor activation in vivo*.* This indicates a removal of the GPCR from the membrane, most probably by internalization. In vitro analyses using markers for membrane, endosomes and lysosome confirmed this hypothesis. This is further supported by the fact that the CAAX::mNG signal remains stable over time indicating that the protein persists at the membrane (Fig. 5, Fig. S5). Internalization is a well-established concept of mammalian NPY receptors (summarized in [55]). We now extend this knowledge to show that also the distant *C. elegans* homolog NPR-11 undergoes receptor internalization. Hence, the concept of internalization is transferable to *C. elegans.* This is a novel finding in the context of the nematode’s GPCR biology. Furthermore, the enhanced bystander BRET measurements proved as a suitable tool for studying this aspect of receptor trafficking. Due to the existence of numerous fluorescent proteins suitable for expression in *C. elegans*, it might be possible to generate a two-step enhanced bystander NanoBRET by tagging two cell compartments with different fluorescent proteins (e.g. membrane and recycling endosomes), which provide a distinguishable spectrum and monitor trafficking of Nluc-fused proteins between different compartments by measuring specific fluorescent signals with a spatial and temporal resolution.

One difficulty of in vivo NanoBRET is to track changes of proteins within the organism as most molecular processes are in steady-state. Specific interactions need a trigger such as ligand-binding to change the equilibrium. Since the components of NanoBRET are mostly proteins, expression can be regulated easily in *C. elegans.* Combining NanoBRET with knockout strains and systems enabling controllable protein expression (for instance through heat-shock promoters [56], the Q system [57], and the auxin-inducible system [58]) offers numerous possibilities of real-time tracking protein interactions and dynamics in vivo*.* In the light of the promising applications of NanoBRET in *C. elegans*, careful evaluation of each set up needs to be taken as the success of NanoBRET depends on the tissue of interest, the subcellular localization of the proteins to be investigated and thus, their accessibility. It also has to be noted that the technique in its current state is not intended to be suitable for high-throughput screenings of interactions, but rather to test distinct interactions and their impact. The tremendous potential of the technique and the extent of its application for intracellular proteins remain to be determined in the future.

## Methods

### Materials and reagents - sources

All standard chemicals were from Sigma Aldrich, ThermoFisher Scientific or Carl Roth GmbH unless stated otherwise. All enzymes were obtained from New England Biolabs. The following reagents, bacterial strains, cell lines and resources were used and respective sources listed:

**Table:** 

REAGENT or RESOURCE	SOURCE	IDENTIFIER
**Bacterial and Virus Strains**		
*E. coli* DH5α	ThermoFisher Scientific	18,258,012
*E. coli* OP50	CGC	N/A
*E. coli* SW106	NCI Frederick	N/A
**Biological Samples**		
*C. elegans* cDNA	This study	
**Chemicals**		
Forskolin	Sigma-Aldrich	CAS 66575–29-9
Sodium azide	Roth	CAS 26628–22-8
Hoechst33342	Sigma-Aldrich	CAS 23491–52-3
DMSO	Sigma-Aldrich	CAS 67–68-5
Lipofectamine 2000	ThermoFisher	#11668019
Trypsin	ThermoFisher	#25300054
HBSS	ThermoFisher	#15356878
HEPES	ThermoFisher	#15630080
MetafectenePro	Biontex	#T040
Coelenterazine H	Prolume/Nanolight	CAS 50909–86-9
LysoTracker Blue DND-22	ThermoFisher	#L7525
**Peptides**		
Peptides	This paper	N/A
**Critical Commercial Assays**		
pGL4.29[luc2P/Hygro/CRE] reporter gene plasmid	Promega	#E8471
ONE-Glo Luciferase Assay System	Promega	#E6120
Nano-Glo® Live Cell Assay System	Promega	#N2011
AlphaScreen cAMP Detection Kit	PerkinElmer	6760635D
**Experimental Models: Cell Lines**		
HEK293	German Collection of Microorganisms and Cell Cultures (DSMZ)	ACC 305
COS-7	German Collection of Microorganisms and Cell Cultures (DSMZ)	ACC 60
**Experimental Models: Organisms/Strains**		
*Caenorhabditis elegans* strains, see Table S3	This paper	N/A
**Oligonucleotides**		
Primers, see Table S2	SeqLab	N/A
**Scientific instruments**		
Plate reader	PerkinElmer/ Tecan	
Micro injector	Eppendorf	
DMi8 confocal microscope	Leica	
Axiovert Observer Z1 microscope	Zeiss	

### Peptide synthesis

Peptides were synthesized by solid-phase peptide synthesis (SPPS) using a Syro II peptide synthesizer (MultiSynTech; resins and amino acid from Iris Biotech) in 15 μmol scale, following the 9-fluorenylmethoxycarbonyl/tert-butyl (Fmoc/tBu) strategy (reviewed in [59]). Briefly, the peptide sequence is built up from C to N terminus on an immobilized solid-phase as a growing peptide chain by repeated steps of coupling N-terminally Fmoc protected amino acid derivatives, and deprotection of the N-terminal Fmoc group to enable the coupling of the next amino acid. Control peptides were generated by scrambling of the peptide sequence and synthesized in parallel. Peptides were cleaved from the resin by incubation with trifluoroacetic acid (TFA)/H_2_O/triisopropylsilane (90/5/5, v/v/v), which simultaneously removes the side chain protection groups from reactive side chains. All peptides were purified to ≥95% homogeneity by preparative HPLC (Shimadzu) using a Phenomenex Aeris, 100 Å (C18) column and linear gradients of solvent B (acetonitrile+ 0.08% trifluoroacetic acid) in A (H_2_O + 0.1% trifluoroacetic acid). The identity of the peptides was confirmed by MALDI-ToF mass spectrometry (Ultraflex III MALDI ToF/ToF, Bruker, Billerica, USA).

To generate the amidated C terminus of FLP-34-1, a TentaGel R RAM resin (Iris Biotech) was used as solid phase. For a fluorescent variant of FLP-34-1, 5(6)-carboxytetramethylrhodamine (TAM) was coupled to the free N-terminus using 2 eq each of the fluorescent dye, diisopropylethylamine (DIEA), and 1.9 eq. 1-[Bis (dimethylamino)methylene]-1H-1,2,3-triazolo[4,5-b] pyridinium 3-oxide hexafluorophosphate (HATU) in dimethylformamide (DMF) at room temperature overnight in the dark. Peptides were then further cleaved from the resin and purified as described above.

LAT-1 peptides (wild-type and scrambled sequence) were fluorescently labeled at the C terminus. For that purpose, 15 μmol TentaGel S HMP resin (Iris Biotech) was loaded with Nα-protected 2,3-diaminoproprionic acid with an orthogonally protected side chain (Fmoc-Dap (Dde)-OH). After automated elongation of the peptide sequence up to the N terminus, Dap (Dde) was selectively deprotected on resin by repeated addition of 2% hydrazine (v/v) in DMF (10 × 10 min), and coupled to a TAM-fluorophore as described above. For non-fluorescent peptide variants, the liberated Dap side chain was acetylated on resin using 10 eq of acetic anhydride (Ac_2_O) and DIEA in dichloromethane (DCM) at room temperature for 15 min. Peptides were further cleaved from the resin and purified as described above.

### *C. elegans* lysis

10 adult wild-type hermaphrodites were transferred into lysis buffer (1x PCR buffer, 2 μg/ml proteinase K), once freeze cracked at − 80 °C for 5 min and lysed at 60 °C for 45 min. Proteinase K was inactivated at 90 °C for 15 min and the mixture was used as DNA template for PCR reactions.

### Generation of constructs

#### NPR-11 constructs fused to a nanoluciferase

##### *Npr-11p::Nluc::npr-11* and *npr-11p::Nluc::npr-11::gfp*

For expressing a variant of the nanoluciferase (Nluc, Promega, USA), fused to the N terminus of *npr-11*, 3 kb upstream of *npr-11* was amplified from fosmid WRM0616cB05 with primer pnpr-11_SbfI_f and pnpr-11_XbaI_r attaching *Sbf*I and *Xba*I restriction sites. The fragment was cloned into two modified pPD95.79 (from A. Fire, Addgene plasmid #1496) expression vectors, one with and one without a GFP, using *Sbf*I and *Xba*I. *npr-11* cDNA was purchased from GenScript and amplified with npr-11_nluc_f and npr-11_XmaI_r/ npr-11_XmaI_GFP_r (for sequences see Table S2) generating an overlap to the Nluc at the 5′ end and an *Xma*I restriction site at the 3′ end. The Nluc with an additional SGGGGS linker at the 3′ end was amplified from pNL1.3_secNluc plasmid (Promega, USA) with primers Nluc_XbaI_f and Nluc_NPR-11_r generating an *Xba*I restriction site at 5′ and an overlap to *npr-11* at the 3′ end. The Nluc and *npr-11* fragments were fused together by overlap PCR and inserted into the modified pPD95.79 with *pnpr-11* using *Xba*I and *Xma*I restriction sides (pSP167 with GFP, pSP168 without GFP). Primer sequences are shown in Table S2.

##### Npr-11p::npr-11::Nluc

For studying intracellular BRET, the Nanoluciferase (Nluc,Promega, USA) was fused to the C terminus of *npr-11*. cDNA of *npr-11* was amplified with XbaI_npr-11_f/npr-11_Nluc_r from pSP168 inserting an *Xba*I restriction site at the 5′ end and parts of a SGGGGS linker at the 3′ end. The Nluc was amplified from pNL1.3_secNluc plasmid (Promega, USA) using Nluc_Linker_npr-11_f/Nluc_XmaI_r to add a 5′ SGGGGS Linker and an *Xma*I restriction site at the 3′ end. Both fragments were fused together via overlap PCR and inserted into a modified pPD95.79 containing 3 kb upstream of *npr-11* (generated as described above) using *Xba*I and *Xma*I restriction sites resulting in pSP185. Primer sequences are shown in Table S2.

#### *Npr-11* constructs for in vitro analysis

For cAMP reporter gene assays, a plasmid was used containing the cDNA of *npr-11* fused C-terminally to the enhanced yellow fluorescent protein (eYFP) in the pVitro2-hygro-mcs vector (InvivoGen) generated previously [23]. An *npr-11* fused to eCFP used for imaging was generated from this construct by PCR-overlap extension, using the primers pVitro_prolong_for and NPR11-Linker_rev to amplify the NPR-11 part, while eCFP was amplified from Y1-eCFP_N1 [60] using the primers Linker-eCFP_for and N1_rev. The genetic fusion *npr-11:eCFP* was then ligated into the pVitro2 vector using the restriction enzymes *EcoR*V and *Xba*I (all enyzmes from ThermoFisher). The cDNA of the membrane marker CAAX::mNG was cloned from the expression plasmid containing synthetic introns (see below) using PCR overlap extension with the primers HindIII_Kozak_mNG_part1_for, mNG_part1_rev, mNG_part2_for, mNG_part2_rev, mNG_part3_for, mNG_part3_rev, mNG_part4-Li-CAAX_for, mNG_part4-CAAX_NotI_XhoI_XbaI-rev, and sub-cloned into an empty pcDNA3 vector using *Hind*III and *Xba*I.

For NanoBRET binding assays, the nanoluciferase (Nluc, Promega, USA) was genetically fused to the N terminus of *npr-11*, spaced by a SGGGGS linker as previously described [35]. To facilitate expression and targeting to the plasma membrane, the Nluc sequence was preceded by a secretion signal derived from human IL-6 as previously described (secNluc [5];). The Nluc sequence was amplified from the pNL1.3_secNluc plasmid (Promega, USA).

#### Rpl-28p::mNeonGreen::CAAX

For a stable mNeonGreen (mNG) localization in all somatic cells on the inner plasma membrane, mNG was fused to the CAAX motif of *C. elegans let-60* separated by a DNA linker. The linker (amino acid sequence: GSAGTMASNNTASG) and the CAAX sequence (amino acid sequence: KPQKKKKCQIM*) were added to mNG amplified from vector pDD346 (from D. Dickinson, Addgene plasmid #133311) by three subsequent overlap PCR with following primers: mNG_XmaI_f (1. - 3.), mNG_CAAX_1_r (1.), mNG_CAAX_2_r (2.) and mNG_CAAX_3_EcoRI_r (3.) attaching unique restriction sides for *Xma*I and *Eco*RI. The resulting construct was cloned upstream of a 1500 bp sequence 5′ of *rpl-28* amplified from pGC185 [61] with primers rpl-28_SbfI_f and rpl-28_XmaI_r into pPD95.79 with *Sbf*I and *Xma*I yielding plasmid pSP177. For primer sequences see Table S2.

#### Lat-1p::lat-1(1–249)::Nluc::lat-1(250–650)::GFP::lat-1(651–1015)

The Nluc was inserted into LAT-1 between the hormone-binding and the GPCR autoproteolysis-inducing domain (GAIN) after amino acid position 249 into vector pSP5 [30], which contains the genomic sequence of *lat-1* with the 7 kb promoter sequence, using recombineering [62]. A recombineering targeting cassette consisting of three parts – a kanamycin resistance gene, the first seven amino acids of the *lat-1* exon 5 and the Nluc – was generated. For this purpose, five different fragments were amplified and fused together using an overlap PCR resulting in construct *lat-1(1)::kanR::lat-1(2)::Nluc::lat-1(3)*. Primer pairs were the following: lat-1_1_f and lat-1_1_r for lat-1(1), kanR_f and kanR_f for the kanamycin resistance cassette, lat-1_2_f and lat-1_2_r for lat-1(2), lat-1_Nluc_f and lat-1_Nluc_r for the Nluc, and lat-1_3_f and lat-1_3_r for lat-1(3). Primer sequences are listed in Table S2. pSP5 was transformed together with the respective fragment into electro-competent *E. coli* SW106 cells expressing λ Red genes that promote homologous recombination [62]. Positive cells were selected via kanamycin resistance yielding plasmid pSP181.

### *C. elegans* strains

*C. elegans* strains were maintained as described in [63]. All strains used in this study are listed in Table S3. Some strains were provided by the CGC, which is funded by NIH Office of Research Infrastructure Programs (P40 OD010440).

### Generation of transgenic *C. elegans* strains

All transgenic strains expressed a stable extrachromosomal array. Those strains were obtained by microinjection of plasmid DNA into the syncytial gonad of young adult hermaphrodites according to [64, 65]. The injection mix contained the DNA of interest (10 ng/μl for plasmids encoding *npr-11*; 1 ng/μl for plasmids encoding *lat-1*), a plasmid carrying a selection marker (pRF4 (*rol-6 (su1006))*: 100 ng/μl [65]; IR98 (hygromycin resistance): 30 ng/μl [66]) and was filled up with pBluescript II SK+ vector DNA (Stratagene) as stuffer DNA to achieve a final concentration of 120 ng/μl. After injection, the worms were left to regenerate at 15 °C for three days and positive progeny was selected. For selection for hygromycin B resistance, worms were kept on NGM plates containing 0.3 mg/ml hygromycin B. F2 individuals stably expressing the co-injection marker were established as line.

### Preparation of *C. elegans* for luminescence detection and BRET measurements

All measurements were conducted using synchronized young adult worms (1 day post L4). Individuals were washed twice with HBSS (Hanks’ balanced salt solution with 25 mM HEPES, pH 7.4, 37 °C) and the number of worms/ μl was calculated.

For luminescence measurements of intact worms, animals (1; 10; 50; 100 or 500) were directly transferred into the wells of a white flat-bottom 384-well plate in 50 μl HBSS buffer. Worms to be cut were pre-washed with M9 + 0.1% Tween to avoid sticking of worms to the dish and subsequently transferred in 2 ml of HBSS. Incisions were made in the middle of the body with a scalpel blade and worms were immediately afterwards transferred with a glass pipette into HBSS buffer on ice. For mechanically cracking, a worm suspension was homogenized in tubes with 150 μl HBSS and six glass beads (Ø = 3 mm) using with a Precellys homogenizer (Bertin instruments, France) for 40 s at 5000 rpm. Samples were placed on ice until further usage.

### Luminescence detection

50 prepared worms were transferred into a white flat bottom 384-well plate in a total volume of 50 μl HBSS + 25 mM HEPES (pH 7.4; denoted BRET buffer). Immediately after adding coelenterazine H solution (Nanolight/ Prolume, USA) to a final concentration of 4 μM, luminescence was detected using an EnVision plate reader (PerkinElmer, USA) and a NanoBRET Blue 460/80 nm filter with 1000 ms integration time (five repeats every 5 min for long time observation).

### In vivo bioluminescence energy transfer (BRET) binding assay

For BRET binding assays in vivo, the nanoluciferase (Nluc) was applied as energy donor and either peptides labeled with the TAM fluorophore or the mNeonGreen fluorescent protein fused to a receptor served as acceptor.

The peptide-receptor binding BRET was conducted with TAM-FLP-34-1/TAM-pLAT-1 and their corresponding scrambled versions dissolved in H_2_O + 0.5% BSA and 5% DMSO. 30 Nluc-expressing worms with incisions in 50 μl BRET buffer were incubated with dissolving buffer or peptide solutions of final concentrations ranging from 0.05–10 μM in a 384-well plate. During a 25 min incubation with gentle shaking, 10 μl Nano-Glo Live Cell Reagent (N2011, Promega, USA) of a 5x stock were added according to manufacturer’s instructions after 15 min to each well. Competition-binding assays were performed similarly, but additionally, unlabeled peptide (final concentrations 0.016–10 μM) was pipetted into each well and 5 μl of a 10x stock Nano-Glo Live cell reagent was applied after 15 min.

The K_i_ of FLP-34-1 was calculated defining TAM-FLP-34-1 as ‘hot’ ligand with c = 1.6 μM and K_d_ = 1.5 μM with a one-site fit K_i_ with GraphPad Prism version 6 (GraphPad Software).

Luminescence (L) and fluorescence (F) were detected with a microplate plate reader (Tecan Spark) using the following filter set: donor = 430–470 nm, acceptor = 550–700 nm with 1000 ms integration time. BRET was calculated by dividing F values by L values. For each experiment, the background signal of donor-only cells was subtracted, such that the BRET ratio at 0 μM ligand equals 0.00. In Fig. 3B (left) and Fig. 3D, the mean of *n* = 4 (NPR-11) or *n* = 3 (LAT-1) independent, individually background-corrected single experiments is presented. The netBRET presented in Fig. 3B (right) is calculated by subtracting the averaged, baseline-corrected values of the scrambled control from the averaged, baseline-corrected BRET values of the active peptide. The K_d_ value of TAM-FLP-34-1 in vivo was calculated using GraphPad Prism 6 version (GraphPad Software) with a one-site total and nonspecific binding fit.

Luminescence levels depended on the used strain, reaching (without ligand) approximately 2000 AU in *Nluc::npr-11* animals and up to 32,000 AU in *Nluc::lat-1* expressing worms. For enhanced bystander BRET analyses, 30 cut worms (APR716 and APR718) in 50 μl HBSS buffer were incubated for 10 min with 10 μl of Nano-Glo Live Cell Reagent (5x stock). Luminescence and fluorescence were measured once prior to peptide stimulation. Afterwards, FLP-34-1 (final concentration 5 μM) or H_2_O with 0.5% BSA and 2.5% DMSO as control were added to each well and luminescence and fluorescence was measured over the course of 80 min. The acceptor filter for this setting was altered to 505–605 nm, while the donor filter remained at 430–470 nm, as before.

### In vitro bioluminescence energy transfer (BRET) binding assay

The binding of TAM-FLP-34-1 and its scrambled analog was also tested in vitro using membranes of transfected HEK293 cells as described previously [35]. Briefly, membranes of HEK293 cells transiently transfected with Nluc::NPR-11::eYFP were prepared analogously to a described protocol [67, 68]. TAM-labeled peptide in a concentration range of 10^− 12^ M to 10^− 5^ M was incubated with the membranes containing 0.5 μg total protein in 90 μl BRET buffer containing 0.1% bovine serum albumin and Pefabloc SC in solid black 96 well plates for 10 min under gentle agitation at room temperature. Directly before the measurements, coelenterazine H in BRET buffer (10x stock solution) was added to a final concentration of 4 μM, and BRET was measured in a microplate reader (Tecan Spark) with the following filter settings: luminescence (L) 430–470 nm, fluorescence (F) 550–700 nm. The BRET ratio was calculated by the ratio of F/L. The K_d_ values were obtained from a three-parameter logistic fit in GraphPad Prism version 5.03 (GraphPad Software).

### Cell culture

All in vitro experiments were carried out using either HEK293 cells (*Homo sapiens*, embryonic kidney, DSMZ (German Collection of Microorganisms and Cell Cultures) ACC 305) or COS-7 cells (African green monkey, *cercopithecus aethiops*, kidney, DSMZ (German Collection of Microorganisms and Cell Cultures) ACC 60). HEK293 cells were kept in Dulbecco’s Modified Eagle’s Medium (DMEM) with Ham’s F-12 (v/v) and 15% (v/v) heat-inactivated fetal calf serum (FCS), while COS-7 cells were cultured in DMEM with 10% (v/v) FCS and 1% (v/v) Penicillin-Streptomycin. All cells were kept at 37 °C under a humidified atmosphere (5% CO_2_).

### cAMP assay

NPR-11 activation was read out in the G_i/o_ pathway by a cAMP reporter gene assay in transiently transfected HEK293 cells as described previously [23]. Briefly, HEK293 cells were grown to 70% confluency and transiently co-transfected with the receptor plasmid (2 μg) and the reporter gene plasmid pGL4.29 [luc2P/CRE/Hygro] (Promega; 2 μg) with MetafectenePro (Biontex). Cells were then re-seeded into 384-well plates. On the next day, the medium was removed, and the cells were stimulated with peptide solution (20 μl) containing 5 μM forskolin (stimulating intracellular cAMP levels) in DMEM and incubated for 4 h. Luciferase substrate OneGlo in lysis buffer (Promega) was added and incubated for 5 min. Luminescence was measured in a microplate reader (Tecan Spark). Data analysis was performed with GraphPad Prism version 5.03 (GraphPad Software) and is presented as x-fold of forskolin.

G_s_-coupling of LAT-1, quantified by detection of intracellular cAMP accumulation, was measured using an AlphaScreen cAMP detection kit (PerkinElmer, USA). Briefly, COS-7 cells (15,000 cells/ well) were transfected in 96-well plates with either 200 ng of LAT-1-encoding plasmid or empty vector using Lipofectamine2000 (ThermoFisher Scientific). 48 h post transfection, the cells were stimulated with 100 μM peptide solution in HBSS containing 1 mM 3-isobutyl-1-methylxanthine (IBMX) and 1% DMSO and control buffer without peptide for 30 min at 37 °C. Subsequently, the medium was displaced with lysis buffer (5 mM HEPES, 0.3% Tween-20, 0.1% BSA, 1 mM IBMX, pH 7.6) and plates were frozen at − 80 °C until further use. The following steps were conducted as stated in the manufactures’ protocol of the AlphaScreen cAMP detection kit (PerkinElmer, USA) and an EnVision plate reader (PerkinElmer, USA) was used to detect the fluorescence. The data were analyzed with GraphPad Prism version 6 (GraphPad Software) are presented in x-fold over the corresponding empty vector control.

### Fluorescence microscopy and image processing

To determine the distribution of TAM-labeled peptides in worms in vivo, confocal fluorescence microscopy was performed. Wild-type worms were anesthetized with 30 mM NaN_3_ and either intact or scratched incubated with 5 μM TAM-FLP-34-1/ TAM-pLAT-1 and their corresponding scrambled versions in H_2_O + 0.5% BSA for 10 min. After having been washed once in H_2_O + 0.5% BSA, worms were mounted on a microscopy slide containing a 2% agar pad and a drop of H_2_O + 0.5% BSA. Images were recorded as stacks with spatial spacing of 2 μm. Fluorescence was tracked with a Leica DMi 8 microscope (model TL LED, 20x/ 0.75 immersions oil objective) and a DPSS 61 Laser (excitation = 561 nm, emission = 566–700 nm) at room temperature. Images were recorded with a Leica DFC9000 GTC camera and corresponding software (Leica Application Suite X). Contrast, brightness and stack overlay were processed using Fiji [69].

Distribution of the CAAX::mNG fusion protein was determined in intact nematodes expressing CAAX::mNG with an argon laser on 488 nm excitation and 493–550 nm emission with the same technique as described above. The same settings were used to image Nluc::NPR-11::GFP and Nluc::LAT-1::GFP.

Localization of NPR-11::eCFP and CAAX::mNG before and after TAM-FLP-34-1 stimulation was determined in HEK293 cells. The cells were grown on 8-well μ-slides (Ibiditreat) to a confluency of 70–80% and transfected with 1 μg vector DNA (4:1 npr11::eCFP over CAAX::mNG) using Lipofectamine2000 (ThermoFisher Scientific) following the manufacturer’s protocol. On the next day, medium was changed to OptiMEM (Invitrogen Life Technologies) for imaging.

Potential co-localization with recycling endosomes was assessed from co-expression of *rab11-eCFP* (kind gift from R. Schülein, Leibniz-Institute of Molecular Pharmacology, Berlin, Germany), by transfecting plasmids encoding NPR-11::eYFP and rab11::eCFP in a 9:1 ratio (total 1 μg vector DNA) using Lipofectamine2000.

Potential co-localization with lysosomes was investigated in cells expressing only NPR-11:eYFP (1 μg vector DNA transfected with Lipofectamine2000 as described above), and lysosomes were stained for 30 min with Lysotracker blue, (Invitrogen) before peptide stimulation.

Cells were examined before, 30 min and 60 min after peptide stimulation (100 nM TAM-FLP-34-1 in OptiMEM), as well as 30 min and 60 min after agonist wash-out (3 × 5 times with warm OptiMEM). Images were acquired at 37 °C using an Axiovert Observer Z1 microscope (with Apotome, Plan-Apochromat 63x/1.40 Oil DIC objective, filter 47 (436(20)/480(40) for eCFP with acquisition time 1000 ms; filter 46 (500(20)/535(30)) for eYFP and mNG with acquisition time 2000 ms; and filter 31 (565(30)/620(60) for TAM with acquisition time 400 ms; Carl Zeiss). Acquisition time was identical in all experiments and all pictures were processed in the same way.

Processed images were stacked in ImageJ [70] and analyzed as follows: Membrane fluorescence in Fig. 5A was measured from 5 cross-sections per cell, expressing both NPR-11::eCFP and CAAX::mNG, using the plugin “Stack -> measure Stack”, which basically reslices the stack and outputs the maximum fluorescence in each channel. Quantification represents the mean of the maximum membrane fluorescence from three independent experiments with each *N* > 10 cells, and five cross sections per cell. Linescans in Fig. 6 were generated from a selected line in the image stack using “Stack -> Reslice” and the fluorescence intensity was then normalized for each channel.

## Supplementary Information

**Additional file 1.** Supplementary material.

## Data Availability

All data generated or analyzed during this study are included in this published article and its supplementary information files.

## Abbreviations

BiFC: Bimolecular fluorescence complementation
BRET: Bioluminescence resonance energy transfer
*C. elegans*: *Caenorhabditis elegans*
DAP: 2,3-diaminoproprionic acid
EC_50_: Half-maximal efficiency
eCFP: Enhanced cyan fluorescent protein
FLP: FMRF-like peptides
FRET: Fluorescence resonance energy transfer
GPCR: G protein-coupled receptor
kanR: Kanamycin resistance
K_d_: Dissociation constant
K_i_: Inhibitory constant
LAT-1: Latrophilin-like 1
mNG: mNeonGreen
Nanoluc: Nanoluciferase
NPR-11: Neuropeptide receptor 11
NPY: Neuropeptide Y
Rab11: - Ras-related protein 11
rpl-28p: Ribosomal promotor 28
scr: Scrambled
TAM: 5(6)-carboxytetramethylrhodamine
