# Supplementary material for: NanoBRET in C. elegans illuminates functional receptor interactions in real time
Source: BMC Mol Cell Biol. 2022 Jan 31;23:8. doi: 10.1186/s12860-022-00405-w (PMC8805316; doi:10.1186/s12860-022-00405-w)
Supplement: Supplementary file 1 — Additional file 1. Supplementary material. [file 12860_2022_405_MOESM1_ESM.zip › Supplementary Material_revised.pdf]

SUPPLEMENTARY MATERIAL

Table S1: Numerical and raw data of Figs. 3B-E, 4B, C, 5B and S3A-C.

Primary data of figure 3B, mean ± SEM of ≤ 3 technical replicates, I – IV define independent experiments								
Log c peptide [M]	Nluc::NPR-11 Luminescence L [AU] mean ± SEM; fluorescence F [AU] mean ± SEM							
	TAM-FLP-34-1				TAM-scrFLP-34-1			
	I	II	III	IV	I	II	III	IV
without	L 1831.5 ± 208.5 F 176.0 ± 32.0	L 1708.7 ± 571.7 F 191.0 ± 64.3	L 1721.0 ± 443.2 F 183.7 ± 25.8	L 2513.3 ± 249.4 F 239.3 ± 13.2	-	L 1858.0 ± 209.6 F 183.7 ± 9.2	L 1028.0 ± 273.7 F 111.3 ± 14.3	L 1609.7 ± 160.6 F 145.0 ± 10.1
-6.80	L 1279.7 ± 388.7 F 136.7 ± 26.0	L 2507.7 ± 418.0 F 256.7 ± 35.4	L 2519.0 ± 244.4 F 260.7 ± 24.0	L 2288.0 ± 179.5 F 242.3 ± 27.5	L 1568.0 F 167.0	L 1666.0 ± 181.8 F 178.0 ± 16.1	L 1229.3 ± 150.1 F 143.7 ± 26.0	L 1385.3 ± 188.5 F 135.3 ± 3.8
-6.30	L 1845.3 ± 511.0 F 196.7 ± 62.7	L 2108.3 ± 376.1 F 245.3 ± 44.8	L 2088.0 ± 159.9 F 234.3 ± 22.0	L 1807.0 ± 138.3 F 209.7 ± 21.4	L 1646.3 ± 131.7 F 167.0 ± 21.0	L 2048.3 ± 362.8 F 218.3 ± 31.7	L 1691.0 ± 251.3 F 186.0 ± 26.8	L 1501.3 ± 93.6 F 163.3 ± 6.8
-5.80	L 1211.7 ± 304.1 F 155.7 ± 43.6	L 2198.3 ± 254.0 F 243.7 ± 22.3	L 2617.7 ± 504.2 F 323.7 ± 48.7	L 1803.7 ± 175.1 F 241.3 ± 12.1	L 1503.3 ± 281.5 F 161.7 ± 26.7	L 1521.0 ± 252.8 F 184.0 ± 30.7	L 2140.7 ± 190.3 F 243.0 ± 18.3	L 1737.7 ± 245.0 F 179.0 ± 26.1
-5.30	L 1054.3 ± 259.8 F 187.3 ± 41.9	L 1863.3 ± 83.8 F 262.0 ± 12.8	L 1801.0 ± 259.2 F 278.3 ± 53.8	L 2205.7 ± 94.0 F 358.7 ± 358.7	L 1614.7 ± 343.9 F 242.7 ± 53.4	L 1999.0 ± 164.5 F 288.0 ± 32.5	L 1469.3 ± 207.9 F 231.3 ± 30.7	L 1443.7 ± 115.9 F 199.3 ± 15.9
-5.00	L 1446.7 ± 439.5 F 269.0 ± 68.4	L 2218.5 ± 514.6 F 371.7 ± 81.6	L 1737.0 ± 183.9 F 321.0 ± 28.9	L 912 ± 109.4 F 190.7 ± 21.2	L 1461.7 ± 104.2 F 241.3 ± 20.7	L 2190.7 ± 405.7 F 364.0 ± 47.3	L 2688.7 ± 405.7 F 516.0 ± 69.3	L 1010.5 ± 253.5 F 166.0 ± 39.0

Primary data of figure 3C, mean ± SEM of ≤ 3 technical replicates, I – IV define independent experiments				
Log c peptide [M]	Nluc::NPR-11 Luminescence L [AU] mean ± SEM; fluorescence F [AU] mean ± SEM			
	FLP-34-1 vs. TAM-FLP-34-1 (1.6 µM)			
	I	II	III	IV
without	L 3079.0 ± 164.5 F 347.7 ± 13.6	L 2420.0 ± 132.7 F 256.0 ± 23.1	L 1747.3 ± 174.2 F 187.7 ± 18.8	- -
-7.3	L 2807.3 ± 368.6 F 298.0 ± 31.2	L 2102.3 ± 109.0 F 228.0 ± 13.6	L 2621.3 ± 717.7 F 314.7 ± 74.1	L 2657.7 ± 406.2 F 310.0 ± 43.8
-6.80	L 2993.3 ± 228.6 F 340.0 ± 24.6	L 2378.7 ± 29.5 F 261.0 ± 9.5	L 2045.7 ± 161.2 F 220.3 ± 21.3	L 2758.3 ± 216.9 F 316.3 ± 21.6
-6.30	L 2607.0 ± 65,4 F 303.0 ± 4,7	L 2326.3 ± 159.4 F 262.7 ± 13.0	L 2158.3 ± 197.0 F 231.0 ± 25.4	L 2327.0 ± 328.3 F 279.3 ± 32.9

-5.80	L 2705.3 ± 234.9<br>F 279.7 ± 41.7	L 2649.3 ± 239.9<br>F 259.0 ± 11.4	L 2898.7 ± 104.0<br>F 313.3 ± 10.5	L 2661.3 ± 644.0<br>F 281.0 ± 52.4
-5.30	L 2440.7 ± 311.7<br>F 270.0 ± 37.3	L 2240.0 ± 311.7<br>F 220.7 ± 8.1	L 2213.7 ± 122.8<br>F 229.3 ± 13.3	L 2818.7 ± 73.8<br>F 322.3 ± 8.4
-5.00	L 2229.7 ± 329.8<br>F 253.0 ± 31.0	L 2154.3 ± 368.7<br>F 201.7 ±	L 1711.0 ± 312.1<br>F 181.3 ± 31.3	L 2380.3 ± 148.9<br>F 240.0 ± 10.7

Primary data of figure 3D, mean ± SEM of ≤ 3 technical replicates, I – III define independent experiments

Log c peptide [M]	Nluc::LAT-1<br>Luminescence L [AU] mean ± SEM; fluorescence F [AU] mean ± SEM					
	TAM-pLAT-1			TAM-scrpLAT-1		
	I	II	III	I	II	III
without	L 23272.5 ± 13084.5<br>F 1865.5 ± 969.5	L 29995.0 ± 6258.0<br>F 2444.5 ± 484.5	L 20514.0 ± 2480.0<br>F 1630.7 ± 178.4	L 23272.5 ± 13084.5<br>F 1865.5 ± 969.5	L 29995.0 ± 6258.0<br>F 2444.5 ± 484.5	L 20514.0 ± 2480.0<br>F 1630.7 ± 178.4
-6.80	-	-	L 20392.3 ± 3854.4<br>F 1648.0 ± 289.5	-	-	L 17976.7 ± 1814.6<br>F 1464.7 ± 127.8
-6.30	L 38244.7 ± 1528.7<br>F 3251.0 ± 110.6	L 32514.0 ± 5335.0<br>F 2667.0 ± 437.5	L 18572.7 ± 2633.5<br>F 1532.7 ± 178.0	L 33323.7 ± 1406.7<br>F 3015.0 ± 103.3	L 40239.3 ± 475.4<br>F 3457.3 ± 27.1	L 15320.7 ± 4089.5<br>F 1326.3 ± 353.3
-5.80	L 33534.3 ± 3071.0<br>F 3199.3 ± 309.7	L 37634.3 0 ± 1785.7<br>F 3418.3 ± 141.1	L 20611.5 ± 13635.5<br>F 1899.5 ± 89.5	L 31162.7 ± 1756.4<br>F 3216.3 ± 244.3	L 43143.0 ± 5017.9<br>F 4233.7 ± 519.1	L 22837.0 ± 1419.6<br>F 2250.3 ± 168.1
-5.30	L 28077.0 ± 1264.0<br>F 3513.0 ± 240.0	L 29156.0 ± 3046.0<br>F 3290.5 ± 287.5	L 16034.3 ± 3974.4<br>F 1874.0 ± 437.2	L 29177.0 ± 3257.0<br>F 3941.5 ± 476.5	L 30516.5 ± 11134.5<br>F 4156.5 ± 1586.5	L 15937.3 ± 2459.4<br>F 2134.7 ± 311.3
-5.00	L 31018.0 ± 5663.0<br>F 4481.0 ± 688.0	L 33328.0 ± 4685.0<br>F 4426.0 ± 577.0	L 16746.7 ± 1806.0<br>F 2284.3 ± 214.9	L 29093.5 ± 5480.5<br>F 5191.0 ± 964.0	L 33689.0 ± 3691.0<br>F 5598.5 ± 1012.5	L 18644.0 ± 2811.9<br>F 3231.7 ± 479.5

Primary data of figure 3E, mean ± SEM of ≤ 3 technical replicates, I – III define independent experiments

Time [min]	Nluc::LAT-1<br>TAM-pLAT-1 (0.5 mM)<br>Luminescence L [AU] mean ± SEM; fluorescence F [AU] mean ± SEM					
	BSA			pLAT-1 (10 mM)		
	I	II	III	I	II	III
60	L 15494.7 ±.1920.4<br>F 1307.3 ± 137.9	L 20877.0 ± 3714.5<br>F 1765.7 ± 293.7	L 32124.7 ± 1521.3<br>F 2679.3 ± 125.4	L 12358.0 ± 2205.4<br>F 981.0 ± 176.3	L 16440.7 ± 479.6<br>F 1341.7 ± 26.3	L 16965.0 ± 3190.0<br>F 1355.7 ± 252.7

Primary data of figure 4B, mean ± SEM of ≤ 3 technical replicates, I – VII define independent experiments

Assay	Luminescence L [AU] mean ± SEM; fluorescence F [AU] mean ± SEM	
	NPR-11::Nluc	NPR-11::Nluc + CAAX::mNG

I	L 7430.0 ± 2145.4	F 2044.0 ± 537.0	L 6151.7 ± 1376.4	F 1926.0 ± 438.5
II	L 4418.3 ± 1157.5	F 1212.3 ± 329.1	L 3964.7 ± 467.9	F 1217.7 ± 244.9
II	L 14902.7 ± 153.7	F 4229.3 ± 82.7	L 8770.3 ± 4726.5	F 3097.7 ± 1759.9
IV	L 6911.0 ± 288.3	F 1932.3 ± 65.3	L 2802.0 ± 970.4	F 985.7 ± 394.4
V	L 3429.7 ± 321.96	F 1124.0 ± 89.2	L 3379.0 ± 1485.1	F 1228.3 ± 491.8
VI	-		L 2124.7 ± 474.2	F 660.7 ± 149.5
VII	-		L 5720.0 ± 336.0	F 1717.0 ± 229.0

Primary data of figure 4C, mean ± SEM of ≤ 3 technical replicates, I – III define independent experiments						
Time [min]	NPR-11::Nluc<br>Luminescence L [AU] mean ± SEM; fluorescence F [AU] mean ± SEM					
	BSA			TAM-scrFLP-34-1 (5 mM)		
	I	II	III	I	II	III
0	L 4654.0 ± 661.8<br>F 1287.3 ± 174.1	L 2310.0 ± 469.0<br>F 641.3 ± 161.1	L 8770.3 ± 4726.5<br>F 3097.7 ± 1759.9	L 3997.3 ± 869.9<br>F 1156.0 ± 257.0	L 1979.3 ± 669.7<br>F 594.0 ± 210.9	L 6426.0 ± 1912.2<br>F 2131.0 ± 663.0
5	L 4873.0 ± 793.8<br>F 1460.7 ± 231.2	L 2186.7 ± 461.0<br>F 677.3 ± 143.3	L 11379.3 ± 4658.6<br>F 3893.3 ± 1780.4	L 3981.3 ± 881.3<br>F 1147.0 ± 277.6	L 2115.3 ± 639.4<br>F 617.3 ± 181.9	L 8977.0 ± 1772.8<br>F 2952.0 ± 640.4
10	L 5492.7 ± 940.5<br>F 14646.0 ± 263.1	L 2257.7 ± 489.4<br>F 644.0 ± 127.0	L 13174.7 ± 4530.5<br>F 4423.3 ± 1675.9	L 4111.7 ± 973.1<br>F 1236.7 ± 307.9	L 2772.0 ± 671.6<br>F 828.0 ± 244.1	L 10269.0 ± 1737.8<br>F 3245.3 ± 668.9
15	L 5910.3 ± 1016.5<br>F 1756.3 ± 224.5	L 2221.3 ± 434.0<br>F 655.3 ± 148.0	L 13769.0 ± 3649.6<br>F 4569.0 ± 1317.7	L 4315.3 ± 1013.1<br>F 1216.3 ± 257.4	L 3207.3 ± 717.0<br>F 913.3 ± 222.0	L 10732.7 ± 1381.2<br>F 3290.3 ± 557.9
20	L 5634.0 ± 938.9<br>F 1729.7 ± 304.8	-	L 14146.0 ± 2822.1<br>F 4643.0 ± 1105.5	L 4234.3 ± 902.2<br>F 1283.3 ± 303.0	-	L 10085.0 ± 1100.5<br>F 3161.7 ± 432.2
25	L 5565.0 ± 801.6<br>F 1542.3 ± 226.9	L 2925.3 ± 419.1<br>F 899.3 ± 134.5	L 14359.7 ± 2074.7<br>F 4641.0 ± 936.3	L 4162.3 ± 890.0<br>F 1198.3 ± 260.3	L 3666.7 ± 871.4<br>F 1014.0 ± 228.8	L 9721.3 ± 887.9<br>F 3062.3 ± 381.7
30	L 5349.0 ± 736.8<br>F 1581.7 ± 230.4	L 3644.0 ± 376.9<br>F 1067.0 ± 97.2	L 13739.3 ± 1734.9<br>F 4349.3 ± 803.7	L 4117.0 ± 747.6<br>F 1198.3 ± 226.8	L 3950.7 ± 885.6<br>F 1148.3 ± 312.5	L 9228.3 ± 783.2<br>F 2905.7 ± 278.5
35	L 5294.0 ± 716.3<br>F 1503.3 ± 200.5	-	L 12936.3 ± 1547.6<br>F 4357.3 ± 704.5	L 3872.7 ± 618.9<br>F 1106.0 ± 197.6	-	L 8740.7 ± 647.4<br>F 2685.3 ± 250.1
40	L 4933.0 ± 645.4<br>F 1451.7 ± 184.8	-	L 11948.7 ± 1421.0<br>F 3851.7 ± 648.7	L 3863.7 ± 620.0<br>F 1103.7 ± 190.8	-	L 8438.7 ± 574.6<br>F 2520.7 ± 222.4
45	L 4657.3 ± 514.3<br>F 1408.0 ± 125.7	L 4371.0 ± 113.4<br>F 1297.0 ± 70.5	L 11358.3 ± 1509.7<br>F 3677.7 ±.613.9	L 3782.7 ± 669.3<br>F 1119.3 ± 186.7	L 3736.7 ± 888.4<br>F 1070.0 ± 239.8	L 8012.00 ± 503.0<br>F 2334.0 ± 182.7

50	L 4584.3 ± 464.1 F ±	L 4454.7 ± 426.1 F 1301.7 ± 112.7	L 10516.3 ± 1256.9 F 3425.7 ± 593.5	L 3792.7 ± 587.5 F 1090.3 ± 212.4	L 3634.0 ± 785.4 F 1040.3 ± 194.6	L 7625.7 ± 464.2 F 2251.0 ± 186.8
55	L 4749.3 ± 503.4 F 1365.0 ± 103.3	L 4654.7 ± 521.1 F 1371.7 ± 174.5	L 9865.7 ± 1242.4 F 3135.3 ± 508.7	L 3716.3 ± 472.6 F 1017.3 ± 117.0	L 3692.7 ± 814.8 F 1071.7 ± 194.6	L 7068.0 ± 412.8 F 2054.7 ± 140.7
60	L 4901.0 ± 633.5 F ±	L 5012.0 ± 827.7 F 1526.7 ± 302.7	L 9238.3 ± 1252.5 F 2904.0 ± 508.7	L 3754.3 ± 479.5 F 1118.7 ± 151.7	L 4077.0 ± 737.5 F 1188.0 ± 225.6	L 6753.3 ± 234.9 F 1936.3 ± 104.8
65	L 4999.7 ± 798.4 F 1525.0 ± 206.1	L 5449.7 ± 806.7 F 1737.7 ± 298.1	L 8560.7 ± 954.9 F 2747.7 ± 478.7	L 3592.3 ± 409.7 F 1039.7 ± 104.8	L 4299.7 ± 742.6 F 1217.0 ± 213.0	L 6338.7 ± 219.9 F 1817.0 ± 66.8
70	L 4954.0 ± 738.6 F 1459.7 ± 221.4	L 6000.7 ± 540.5 F 1846.3 ± 199.5	L 8149.7 ± 1013.9 F 2504.0 ± 391.0	L 3359.7 ± 336.0 F 972.7 ± 115.6	L 4339.0 ± 769.4 F 1237.7 ± 208.6	L 5972.3 ± 249.2 F 1755.0 ± 94.2
75	L 4922.0 ± 880.6 F F 1435.3 ± 244.2	L 5881.7 ± 388.9 F 1854.3 ± 178.4	L 7520.7 ± 1001.4 F 2345.0 ± 450.7	L 3298.7 ± 411.9 F 990.0 ± 124.0	L 4272.3 ± 704.4 F 1253.7 ± 171.2	L 5587.3 ± 215.9 F 1622.7 ± 55.3
80	L 4690.7 ± 762.0 F 1354.7 ± 209.3	-	L 7100.7 ± 834.7 F 2155.3 ± 322.5	L 3282.7 ± 351.0 F 964.7 ± 99.7	-	L 5358.3 ± 189.1 F 1556.7 ± 68.1

Primary data of figure 5B, mean ± SEM of 3 independent assays

construct	Membrane fluorescence [AU] + C.I. [Lower – Upper] Time after stimulation				
	0 min	30 min	60 min	Agonist wash-out + 30 min	Agonist wash-out + 60 min
NPR-11::CFP	47.0 [44.3 - 49.6]	47.0 [43.3 – 50.7]	39.6 [36.0 – 43.2]	43.0 [40.2 – 45.7]	41.7 [38.4 – 45.1]
CAAX ::mNG	35.0 [31.6 – 38.5]	39.8 [34.4 – 45.3]	39.6 [33.5 – 45.6]	40.1 [35.1 – 45.1]	32.4 [29.4 – 35.3]

Primary data of figure S3A, mean ± SEM of ≤ 3 technical replicates, I – II define independent experiments

Log c peptide [M]	Nluc::NPR-11 Luminescence L [AU] mean; fluorescence F [AU] mean			
	TAM-FLP-34-1		TAM-scrFLP-34-1	
	I	II	I	II
without	L 380638.3 F 23667.7	L 469240.7 F 29168.3	L 323347.0 F 20302.0	L 428056.3 F 26897.7
-9,8	L 339953.0 F 21773.3	L 400754.3 F 25580.3	L 309990.0 F 19386.0	L 397071.3 F 24693.3
-8,8	L 339957.7 F 23720.7	L 402603.0 F 28164.0	L 311779.0 F 19459.3	L 409736.7 F 25778.3
-8,3	L 344593.7 F 25665.7	L 407620.7 F 30328.3	L 317827.7 F 19953.0	L 406932.0 F 25612.7
-7,8	L 337151.3 F 27506.3	L 399637.3 F 32326.3	L 310546.3 F 19533.0	L 407883.3 F 25585.0

-7,3	L 324039.0<br>F 34200.3	L 388152.7<br>F 40576.3	L 311015.7<br>F 19502.3	L 407381.0<br>F 25458.0
-6.80	L 310521.7<br>F 47319.7	L 369564.0<br>F 55461.7	L 311208.7<br>F 19766.3	L 402490.3<br>F 25630.0
-6.30	L 279092.3<br>F 63694.7	L 330883.3<br>F 75727.0	L 315503.7<br>F 20297.7	L 395422.3<br>F 25363.7
-5.80	L 250488.0<br>F 75311.7	L 303646.7<br>F 91204.0	L 314729.7<br>F 22118.3	L 413383.7<br>F 29033.7
-5.30	L 236820.3<br>F 80921.3	L 286582.7<br>F 97750.7	L 241369.0<br>F 21564.3	L 280704.3<br>F 24906.0

Primary data of figure S3B, x-fold of forskolin pre-stimulation of two independent assays

Log c peptide [M]	NPR-11		
	Luminescence [x-fold of forskolin]		
	FLP-34-1	TAM-FLP-34-1	TAM-scrFLP-34-1
without	1 ± 0	1 ± 0	1 ± 0
-11	0.899 ± 0.019	0.875 ± 0.025	0.963 ± 0.003
-10	0.873 ± 0.034	0.786 ± 0.068	1.012 ± 0.030
-9	0.612 ± 0.090	0.352 ± 0.109	1.171 ± 0.106
-8	0.296 ± 0.064	0.156 ± 0.065	1.210 ± 0.072
-7,5	0.196 ± 0.041	-	1.119 ± 0.029
-7	0.093 ± 0.054	0.051 ± 0.044	1.181 ± 0.079
-6	0.025 ± 0.045	0.060 ± 0.032	1.010 ± 0.053
-5	0.070 ± 0.035	0.038 ± 0.036	1.010 ± 0.024

Primary data of figure S3C, mean ± SEM of 5 independent assays

plasmid	LAT-1<br>cAMP conc. mean [nM] ± SEM				
	DMSO	plat-1	scrplat-1	TAM plat-1	TAM scplat-1
empty vector	5.39 ± 2.15	2.95 ± 0.77	3.26 ± 0.67	2.86 ± 0.60	4.45 ± 0.71
LAT-1	4.66 ± 1.72	3.73 ± 0.96	3.31 ± 0.66	3.69 ± 0.70	4.63 ± 0.81

Table S2: List of primers used in this study.

Primer	Sequence 5'-3'
rpl-28_SbfI_f	CATGCCTGCAGGTCTGCAGTTTGTGCAAC
rpl-28_XmaI_r	CATCCCGGGGCACGAGAGCGTCGGATATTTTACC
mNG_XmaI_f	GTGCCCCGGGATGGTCTCCAAGGGAGAGGAGG
mNG_CAAX_1_r	GGTGTTGTTGCTCGCCATGGTGCCCGCGCTGCCCTTGTAGAGCTCGTCCATTCC
mNG_CAAX_2_r	CTTCTTCTTCTTTTGTGGCTTGCCGCTCGCGGTGTTGTTGCTCGCCATGG
mNG_CAAX_3_EcoRI_r	GTTGGAATTCTCACATTATTTGACACTTCTTCTTCTTTTGTGGC
lat-1_1_f	TCTGTTCGATGACGCGTGTCCAAT
lat-1_1_r	GCATACATTATACGAAGTTATAAAAGCAGAGTCATAACTC
kanR_f	GAGTTATGACTCTGCTTTTATAACTTCGTATAATGTATGC
kanR_r	GCAAAATCATTTCCATCACAATGAATGTGATAACTTCGTATAGCATAC
lat-1_2_f	GTATGCTATACGAAGTTATCACATTCATTGTGATGGAAATG
lat-1_2_r	GAGCCTCCGCCACCACTACCACTCAAAACACTATTTC
lat-1_Nluc_f	AGTGGTGGCGGAGGCTCAGTCTTCACACTCGAAGATTTCG
lat-1_Nluc_r	ACTACCTCCACCGCCTGACG
lat-1_3_f	CAGGCGGTGGAGGTAGTGTTATTTCGAGTGAGGATG
lat-1_3_r	GTGTCCTCGTACATCCATCAAAACA
pnpr-11_SbfI_f	ATGCCTGCAGGTGGTAGGTTTAGGA
pnpr-11_XbaI_r	CCATTCTAGATTGTTGAAAGTTCTGAAAAT
npr-11_nluc_f	CAGGCGGTGGAGGTAGTGGATCGGTGAATGAATCA
npr-11_XmaI_GPF_r	GTCCCGGGATCAGCTGCTC
npr-11_XmaI_r	GTCCCGGGTCACAGCTGCT
Nluc_XbaI_f	CAATCTAGAATGGTCTTCACACTCGAAG
Nluc_NPR-11_r	ACTACCTCCACCGCCTGACG
npr-11_XbaI_f	CAATCTAGAATGGGATCGGTGAATGAATC
npr-11_nluc_r	CCTCCGCCACCACTCAGCTGCTCATCTTGCTCC
Nluc_Linker_npr-11_f	GAGCAGCTGAGTGGTGGCGGAGGCTCAATGG
Nluc_XmaI_r	GGTCCCGGGTCACGCCAGAATGCGTTCGCACAGC
pVitro_prolong_for	CCCCTTGAGTTTTGAGCGGAGC
NPR11-Linker_rev	GCTCACGACCGGTGGATCCAGCTGCTCATCTTGCTCC
Linker-CFP_for	GATCCACCGGTCGTGAGCAAGGGCGAGGAGCTGTTCAC
N1_rev	CAGGGGGAGGTGTGGGAGG
HindIII_Kozak_mNG_part1_for	TTAAGCTTGCCACCATGGTCTCCAAGGGAGAGGAGGACAACATGG
mNG_part1_rev	GGAGAATTGGAGGTCTCCCTTGGTGGACTTGAGGTTG
mNG_part2_for	CAACCTCAAGTCCACCAAGGGAGACCTCCAATTCTCC
mNG_part2_rev	CCTTGACTTGGGCCTCTCCCTTGATGTGGGATCCCTCG
mNG_part3_for	CGAGGGATCCCACATCAAGGGAGAGGCCCAAGTCAAGG
mNG_part3_rev	CCACTTGAAGGTGGAGATGATGGTCTTGTCGTTTGGGTAGGTCTTC
mNG_part4-Li-CAAX_for	GAAGACCTACCCAAACGACAAGACCATCATCTCCACCTTCAAGTGG
mNG_part4-CAAX_NotI_XhoI_XbaI-rev	CCTCTAGACTCGAGCGGCCGCTCACATTATTTGACACTTC

Table S3: List of *C. elegans* strains used in this study.

Strains	Genotype	Referred to as	Source	Origin
N2	*C. elegans* wild isolate		CGC	Brenner, 1974
RB799	*C25G6.5 npr-11 (ok594) X*		CGC	*C. elegans* Gene Knockout Consortium
APR680	*npr-11 (ok594) X; aprEx268 [nprp-11::Nluc::npr-11::GFP HygR pbluescript]*	*Nluc::npr-11::GFP*	-	This study
APR683	*npr-11 (ok594) X; aprEx271 [nprp-11::Nluc::npr-11 HygR pbluescript]*	*Nluc::npr-11*	-	This study
APR716	*npr-11 (ok594) X; aprEX292 [nprp-11::npr-11::Nluc HygR pBluescript]*	*npr-11::Nluc*	-	This study
APR718	*npr-11 (ok594) X; aprEX294 [nprp-11::npr-11::Nluc prpl-28::CAAX::mNG HygR pBluescript]*		-	This study
APR686	*lat-1(ok1465) II; aprEX273 [latp-1:: lat-1(1-249)::Nluc::lat-1(250-650)::GFP::lat-1(651-1015)], rol-6 (su100); pbluescript]*	*Nluc::lat-1*	-	This study

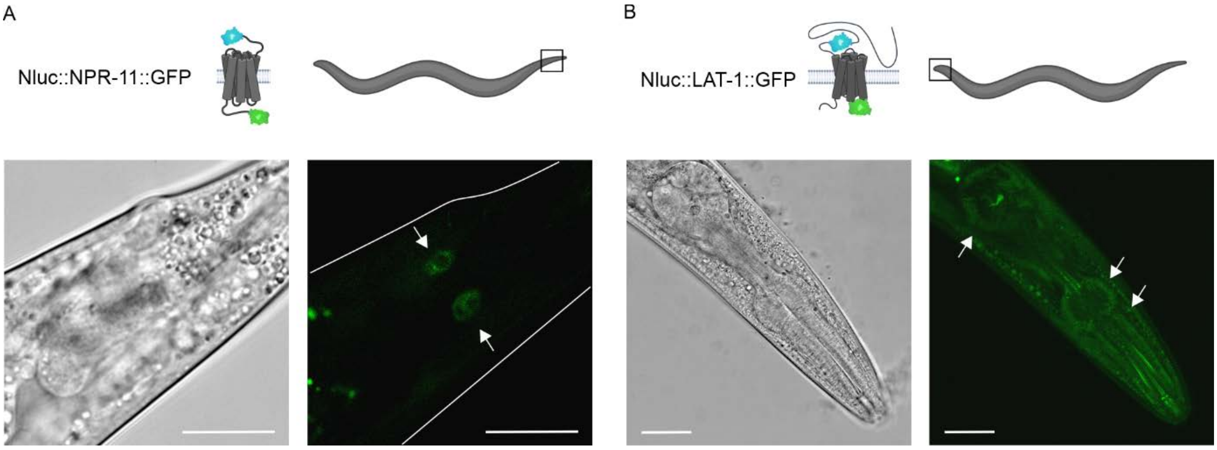

Figure S1: Expression of *Nluc::npr-11* and *Nluc::lat-1* fused to GFP in young adult hermaphrodites. A Expression of *Nluc::npr-11::GFP* was limited to two cells, supposable neurons, within the tail (white arrows). **B** Green fluorescence of Nluc::LAT-1::GFP was found all around the muscular pharyngeal membrane. Fluorescent microscopy images are combined z-stacks with spatial spacing of 1 µm. Scale bars = 25 µm.

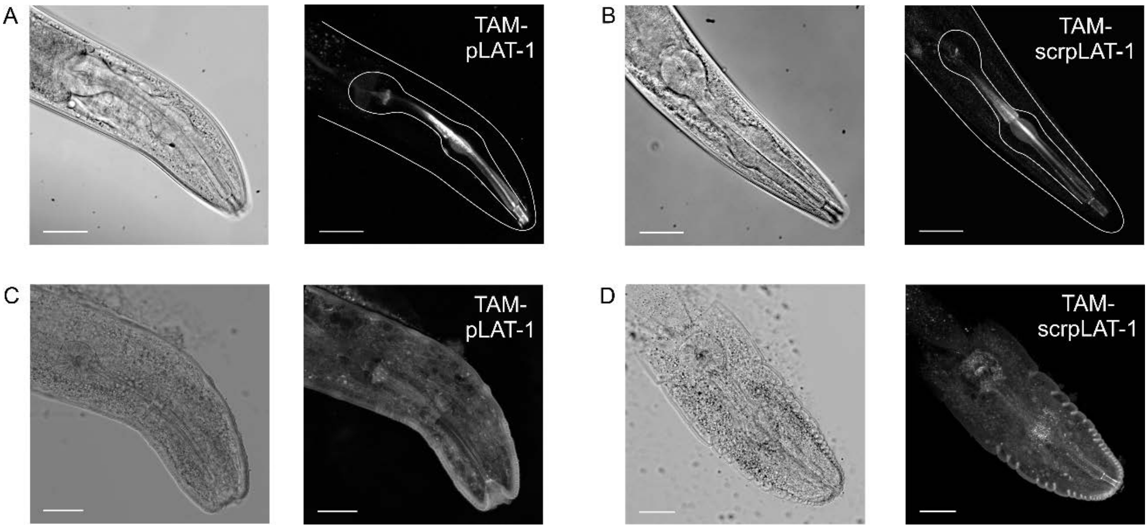

Figure S2: Accessibility and distribution of TAM-pLAT-1 and TAM-scrpLAT-1 in the head of young adult wild-type hermaphrodites. A, B Distribution of fluorescent tetramethylrhodamine (TAM)-labeled peptides (5 µM) in the heads of intact nematodes after 10 min incubation. TAM-pLAT-1 (**A**), and the corresponding scrambled (scr) peptide, TAM-scrpLAT-1 (**B**) show a similar pattern of distribution. Left: DIC; right: fluorescence image. Fluorescence is only localized within the pharynx lumen. **C, D** Distribution of fluorescent TAM-labeled peptides (5 µM) in the heads of nematodes with an incision after 10 min incubation. TAM-pLAT-1 distributes evenly in the heads of wild-type worms with incisions, no distinct structures are stained (**C**). The distribution of TAM-scrpLAT-1 is indistinguishable from the one of TAM-pLAT-1 (**D**). The integrity of the worms in **C** and **D** is damaged due to the preparation affecting the appearance of all structures. Images are combined z-stacks with spatial spacing of 1 µm. Scale bars = 50 µm.

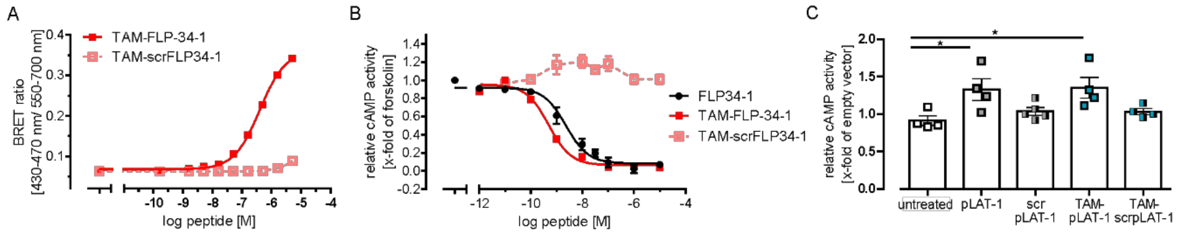

Figure S3: Activity and binding of FLP-34-1 derivatives on NPR-11 and activity of pLAT-1 versions on LAT-1. A BRET binding assays were performed on membrane preparations of HEK293 cells transfected with the *Nluc::npr-11*-encoding plasmid to determine the binding affinities of TAM-FLP-34-1 and TAM-scrFLP-34-1 to Nluc::NPR-11. TAM-FLP-34-1 binds to Nluc::NPR-11 with a K_d value of 388 nM and a ΔBRET of 0.29 ± 0.01, while TAM-scrFLP-34-1 does not generate a measurable BRET signal up to a concentration of 10 µM. For raw luminescence and fluorescence values see Table S1. **B** To test for the functionality of TAM-labelled FLP-34-1 to activate NPR-11, a cAMP reporter gene assay was performed in transfected HEK293 cells. Activated receptors lead to a decrease in the forskolin-induced cAMP levels, and subsequently, the production of the luc2P luciferase, which is controlled by a cAMP-response element. Unlabeled FLP-34-1 activates NPR-11 (EC_{50} = 2.3 nM). Labeling with TAM does not grossly affect the peptide´s activity and TAM-scrFLP-34-1 does not activate NPR-11. Numerical values are given in Table S1.**C** cAMP accumulation assay to determine the activity of pLAT-1-derived peptides labeled with TAM. COS-7 cells transfected with the plasmid encoding *lat-1* were stimulated with pLAT-1 (100 µM). pLAT-1 is able to increase intracellular cAMP levels up to 50 % over untreated controls (DMSO), while scr-pLAT-1 of the same concentration has no activity. Peptide activity is not affected by TAM.

Shown is the mean ± SEM of n ≥ 2 independent assays, each performed in triplicates. * $p \leq 0.05$ compared to untreated DMSO, one-way ANOVA, Dunnett corrected. Non-normalized values are given in Table S1.

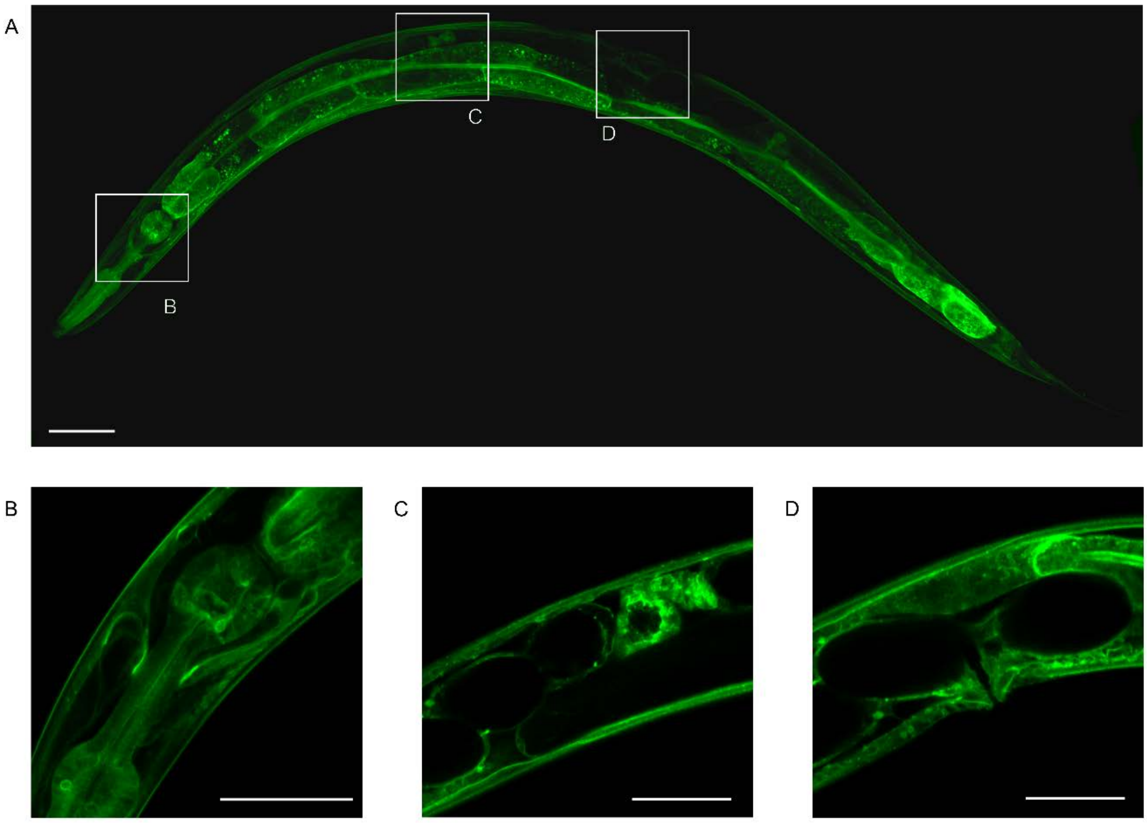

Figure S4: Expression of mNeonGreen fused to the CAAX motif in a young adult nematode. Representative confocal z-stack projection of a young adult nematode expressing mNeonGreen fused to the CAAX motif, which is used as membrane marker. Green fluorescence is present almost in all cells except for gonads and embryos due to the use of a ubiquitous ribosomal promotor. Complete nematode (**A**) and enlarged of the terminal pharyngeal bulb (**B**), spermathecal and part of the gonad (distal end, **C**) and vulva with embryos (**D**). Images are combined z-stacks with spatial spacing of 1 µm. Scale bars = 50 µm.

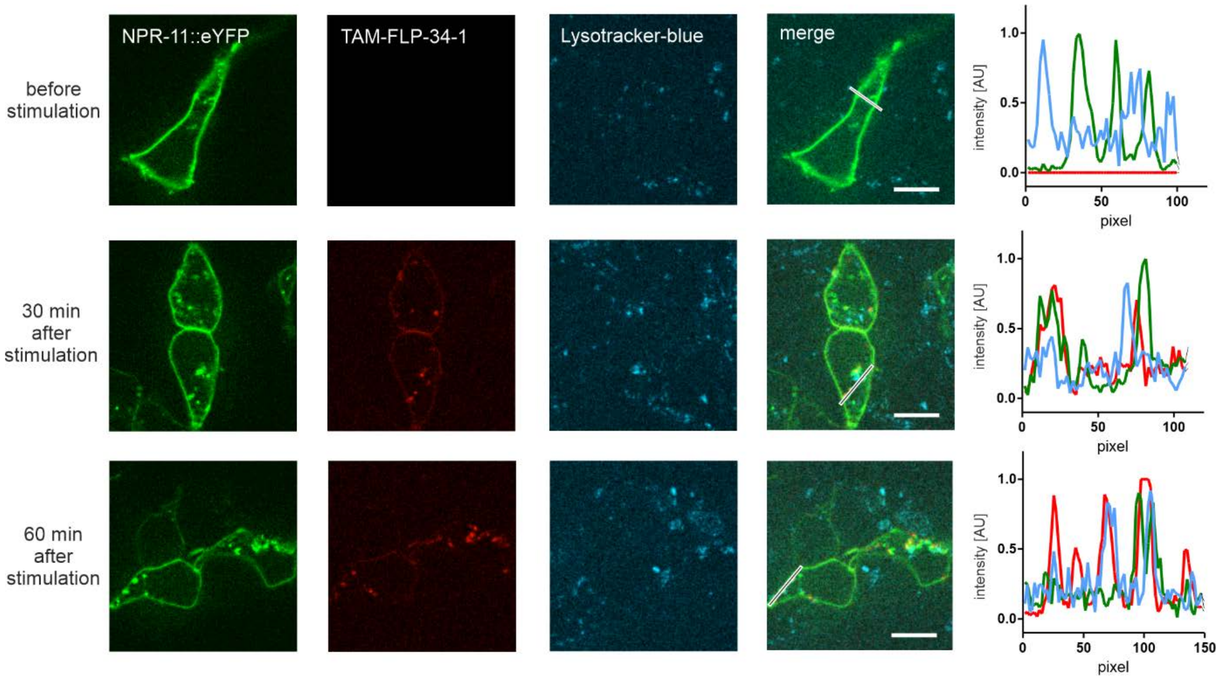

Figure S5: Co-localization of NPR-11::eYFP with lysosomes before and after peptide stimulation. NPR-11 fused to an enhanced yellow fluorescent protein (eYFP) was expressed heterologously in HEK293 cells. Subcellular localization was monitored before and after stimulation with 100 nM TAM-FLP-34-1. Lysosomes were stained with a lysosomal stain (lystrocker blue). The panels on the right show the quantification of the fluorescence intensities across the section indicated in the merged images (white line). Scale bars = 10 µm.

Supplementary References

Brenner, S. 1974. The genetics of Caenorhabditis elegans. Genetics. 77:71–94.
